# D3PM: a comprehensive database for protein motions ranging from residue to domain

**DOI:** 10.1186/s12859-022-04595-0

**Published:** 2022-02-14

**Authors:** Cheng Peng, Xinben Zhang, Zhijian Xu, Zhaoqiang Chen, Yanqing Yang, Tingting Cai, Weiliang Zhu

**Affiliations:** 1 CAS Key Laboratory of Receptor Research, Drug Discovery and Design Center, Shanghai Institute of Materia Medica, Chinese Academy of Sciences, 555 Zuchongzhi Road, Shanghai, 201203 People’s Republic of China; 2 University of Chinese Academy of Sciences, No. 19A Yuquan Road, Beijing, 100049 People’s Republic of China; 3 Open Studio for Druggability Research of Marine Natural Products, Pilot National Laboratory for Marine Science and Technology (Qingdao), 1 Wenhai Road, Aoshanwei, Jimo, Qingdao, 266237 People’s Republic of China

**Keywords:** Protein motion, Motion pattern, D3PM database, Amino acids preference

## Abstract

**Background:**

Knowledge of protein motions is significant to understand its functions. While currently available databases for protein motions are mostly focused on overall domain motions, little attention is paid on local residue motions. Albeit with relatively small scale, the local residue motions, especially those residues in binding pockets, may play crucial roles in protein functioning and ligands binding.

**Results:**

A comprehensive protein motion database, namely D3PM, was constructed in this study to facilitate the analysis of protein motions. The protein motions in the D3PM range from overall structural changes of macromolecule to local flip motions of binding pocket residues. Currently, the D3PM has collected 7679 proteins with overall motions and 3513 proteins with pocket residue motions. The motion patterns are classified into 4 types of overall structural changes and 5 types of pocket residue motions. Impressively, we found that less than 15% of protein pairs have obvious overall conformational adaptations induced by ligand binding, while more than 50% of protein pairs have significant structural changes in ligand binding sites, indicating that ligand-induced conformational changes are drastic and mainly confined around ligand binding sites. Based on the residue preference in binding pocket, we classified amino acids into “pocketphilic” and “pocketphobic” residues, which should be helpful for pocket prediction and drug design.

**Conclusion:**

D3PM is a comprehensive database about protein motions ranging from residue to domain, which should be useful for exploring diverse protein motions and for understanding protein function and drug design. The D3PM is available on www.d3pharma.com/D3PM/index.php.

**Supplementary Information:**

The online version contains supplementary material available at 10.1186/s12859-022-04595-0.

## Background

The conformational diversity of protein is rooted from its structure and is often a key feature of its function [1, 2]. A fundamental recognition of how protein works therefore requires knowledge of its structure and dynamism, which is also helpful to drug discovery and development. For instance, an ensemble docking strategy that tries to solve the problem of receptor flexibility has received increasing attentions on virtual screening [3, 4]. Such conformational diversity can be studied in various ways. X-ray crystallography and nuclear magnetic resonance (NMR) are versatile experimental techniques to obtain biomolecular structures [5, 6]. In computational methods, normal mode analysis and molecular dynamics can be used to predict the conformational diversity of protein [7]. With more and more available protein structures, there is an increasing interest to relate protein structure to motion for studying its function.

As summarized in a number of reviews, most studies about the protein motion have focused on the hinge and shear motions of protein domain [8–11]. Several techniques [12–15] applied to detect dynamical protein domains have been developed such as the difference-distance method and deformation-plot analysis, and a catalog of domain motion types has been complied. Databases of protein domain motions have been also available in recent years, for example the DynDom database [16–19]. In addition, the information of protein motions collected in recent databases involves from small loop to entire subunit besides domain region. However, in many cases, proteins have no obvious domain movement under different conditions, but show significant side-chain motion of binding pocket residue or catalytic residue [20, 21]. The side-chain motion was found to play a crucial role in responding to the access, regiospecificity, stabilization and dissociation of ligand [22, 23]. For example, the most pronounced conformational change simply occurs on the F194 of KAI2 protein with a ~ 90° flip of its benzene ring when bound with inhibitor KAR_1_ [24]. Furthermore, the dynamic residue may impact the conformation of its neighboring region [25]. Therefore, it is of significance to study side-chain motions of the residues within binding pocket.

The protein data bank (PDB) [26] contains nearly 167,000 protein entries (July 2020), and the number is growing at an exponential rate. It is therefore a useful resource for studying protein motions. Three-dimensional (3D) structures of protein are provided in the PDB, but entries of protein are redundant for structures determined under different conditions. Lots of effort has gone into collecting and analyzing the vast amount of data in the PDB, leading to many databases. The MolMovDB [18] is a dominant database containing the information of protein conformational changes. Other databases, such as the ComSin [27], AH-DB [28], PDBFlex [29], and PSCDB [30] provide structural pairs of protein in bound and free states to explore protein motions induced by ligand binding, among which the AH-DB contains the most entries (> 700,000). However, protein motions are sophisticated, which are related not only to its intrinsic flexibility or experimental conditions (such as temperatures and pH), but also to external perturbations like ligand binding [31, 32]. The PCDB [33] and CoDNaS [34] provide redundant structural clusters of protein under different experimental protocols, but the PCDB has been not available for a while. The CCProf [35] is another conformational diversity database that contains 986,187 structural pairs of protein before and after ligand binding, and ten biological features are introduced in the CCProf for studying binding site dynamics. However, it is difficult to study the local motion of binding pocket residue by using these available databases.

In this study, we constructed a database that covers all kinds of protein motions ranging from overall structure to local residue, namely D3PM. The motion patterns in the database are classified into 4 types of overall structural changes and 5 types of pocket residue motions. Considering that the form of structural pairs is more convenient to analyze motion features than that of structural clusters, all the protein motions were provided with structural pairs in the D3PM. We hope that the D3PM will be helpful to explore diverse protein motions and promote the drug discovery and development.

## Construction and content

### The D3PM database construction

All the X-ray structures with resolution better than 3.0 Å were downloaded from the PDB (25th October 2018 for the initial version, 11th April 2021 for the first update), and were divided into pairs of identical proteins that have the same UniProt ID. The oligomerization state was limited to either monomer or homo-multimer to exclude the influence of protein–protein interactions on structural changes. Many of small molecules bound into proteins are crystallographic additives (PEG, etc. Additional file 1: Table S1), and they were manually removed from the protein–ligand complexes. Finally, protein structural pairs were divided into two subsets with the threshold of 2.0 Å for overall C_α_ root mean square deviation (RMSD), which was often used as a threshold for drug discovery [36, 37]. There are redundant protein pairs of identical type of motions. Therefore, a typical protein pair with the most significant motion was selected for each type of motion to construct a non-redundant, contrastive, and classified protein motion database.

**Fig. 1:**
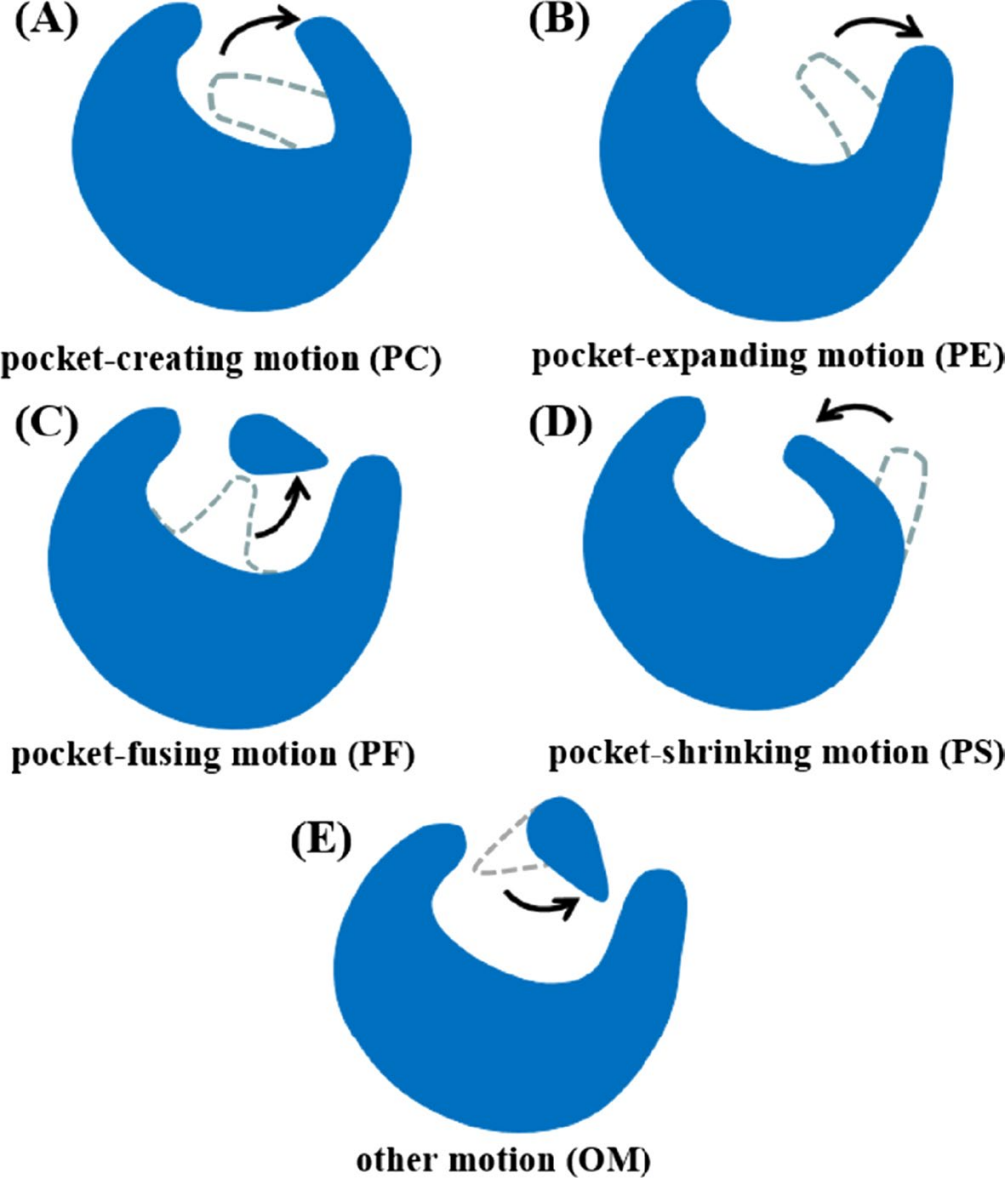
Five classes of pocket residue motions. **A** Pocket-creating motion (PC), **B** pocket-expanding motion (PE), **C** pocket-fusing motion (PF), **D** pocket-shrinking motion (PS), and **E** other motion (OM)

**For protein pairs with overall RMSD that is smaller than 2.0 Å** Although the overall RMSD that is smaller than 2.0 Å indicates similar structures of a protein pair, distinct motions of a few residues within ligand binding site remind us the deficiency of the overall RMSD that it may hide local motions. To explore how pocket residue moves in responding to ligand binding, we firstly calculated the RMSD matrix of each residue around ligand by 5.0 Å for protein pairs of apo and ligand-bound (holo) structures. As observed by Rebecca et al., the protein dynamics could lead to the opening, closing and adaptation of binding pocket, resulting in the appearance/disappearance of a sub-pocket or an allosteric pocket and the pocket breathing motion [38]. To further analyze the motion of pocket residues upon ligand binding, we calculated the pocket volume using the D3Pockets (www.d3pharma.com/D3Pocket/index.php). It is well-known that one type of residue motion can dramatically regulate the “on” and “off” states of binding pocket, which is also called ‘gatekeeper’ [39]. For example, the R410 is the gatekeeper of the adenosine-binding site of NIK (NF-κB-inducing kinase) [40]. Noticeably, most of residue motions simply expand the space of binding pocket. A major reason of the expanding is the moving outwards of pocket residues. On the other hand, the fusion of more than two small sub-pockets provides a large space for ligand binding. On the contrary, to stabilize bound ligand or to take part in catalytic process, pocket residues need to approach the ligand, resulting in a shrinking of binding pocket. For example, the F293 in *apo* FOX-4 cephamycinase moves 2.5 Å inwards upon ligand binding, forming a putative T-shaped π-stacking interaction with the substrate [41]. The rest of residue motions, other than the above four types, have little effect on the space of binding pocket but form better interactions with ligands. Consequently, the residue motions could be classified into five classes (Fig. 1): (a) pocket-creating motion (PC), (b) pocket-expanding motion (PE), (c) pocket-fusing motion (PF), (d) pocket-shrinking motion (PS) and (e) other motion (OM). Each class is represented by a code of two characters: for instance, PC stands for ‘pocket-creating motion’. Finally, we collected a typical pair of the same type of motions with the largest residue RMSD in the D3PM.

Similarly, we calculated the RMSD matrix of pocket residues for protein pairs of holo structures, including the pairs bound with different ligands and the pairs bound with the same ligand. For the pairs bound with different ligands, there are 2,176,460 pairs with at least one residue’s RMSD that is greater than 2.0 Å. We then selected a typical pair with the largest RMSD, and obtained a final set of 1183 cases, viz., 793 pairs with the same ligand binding pocket and 390 pairs with different ligand binding pockets. The 390 cases could be regarded as protein pairs of apo and holo structures, which could also be classified into the five classes (PC, PE, PF, PS, and OM). For those pairs bound with the same ligand, a final set of 1465 cases was selected from pairs with at least one residue’s RMSD that is greater than 2.0 Å.

**For protein pairs with overall RMSD that is greater than 2.0 Å** In this set, protein conformational change may result from both the inherent flexibility and external perturbations like ligand binding. Consequently, to explore how those motions occur, we classified the protein pairs with overall RMSD that is greater than 2.0 Å into four parts: (a) pairs of apo structures, (b) pairs of apo and holo structures, (c) pairs of holo structures with different ligands, (d) pairs of holo structures with the same ligand. For the pairs of apo structures, the inherent flexibility of protein contributes mostly to their conformational changes, since we have excluded the influence of protein–protein and protein–ligand interactions. These datasets containing apo-holo pairs and pairs of holo structures bound with different ligands should be a useful resource to evaluate the protein motions induced by ligands. There are 1111 protein pairs bound the same ligand with RMSD that is greater than 2.0 Å, which should result mainly from the inherently flexibility of protein–ligand complex. For the pairs of holo structures, we calculated the RMSD matrix of the pocket residues, and found that 125 apo-holo pairs have obvious pocket residue motions, which could also be classified into the five classes (PC, PE, PF, PS, and OM).

Finally, the D3PM collects 7649 proteins with overall motions and 3513 proteins with pocket residue motions, as shown in Table 1.

**Table 1:** Summary of the data available in the D3PM (update on 11th April 2021)

The overall protein motions	Number of protein	Overall RMSD (Å)	
		Mean	Maximum
apo & apo	3684	5.31	54.72
apo & holo	1643	4.68	39.53
holo & holo (different ligands)	1211	4.78	63.03
holo & holo (the same ligand)	1111	5.06	63.03

The pocket residues motions	Number of protein	Pocket RMSD (Å)	
		Mean	Maximum
apo & holo	1255	1.91	8.64
holo & holo (different ligands)	793	1.76	8.62
holo & holo (the same ligand)	1465	1.10	9.30

The “apo” referred to as ligand-free protein, and the “holo” referred to as ligand-bound protein. The mean and maximum values were calculated with those representative pairs collected in the D3PM

### Linkage of the D3PM and DrugBank databases

The DrugBank is a free web resource containing comprehensive drugs information with their targets, which greatly facilitates the drug discovery and development [42]. To make full use of protein motions for drug discovery, druggable targets in the DrugBank database are annotated in the protein motion list in the D3PM. For example, the target carbonic anhydrase 2 (PDB ID: 3HS4) can be found with three kinds of motions in the D3PM, including overall conformational changes caused by its inherent flexibility and ligands binding, and PE type of pocket residue motion.

## Utility and discussion

### User interface

**Fig. 2:**
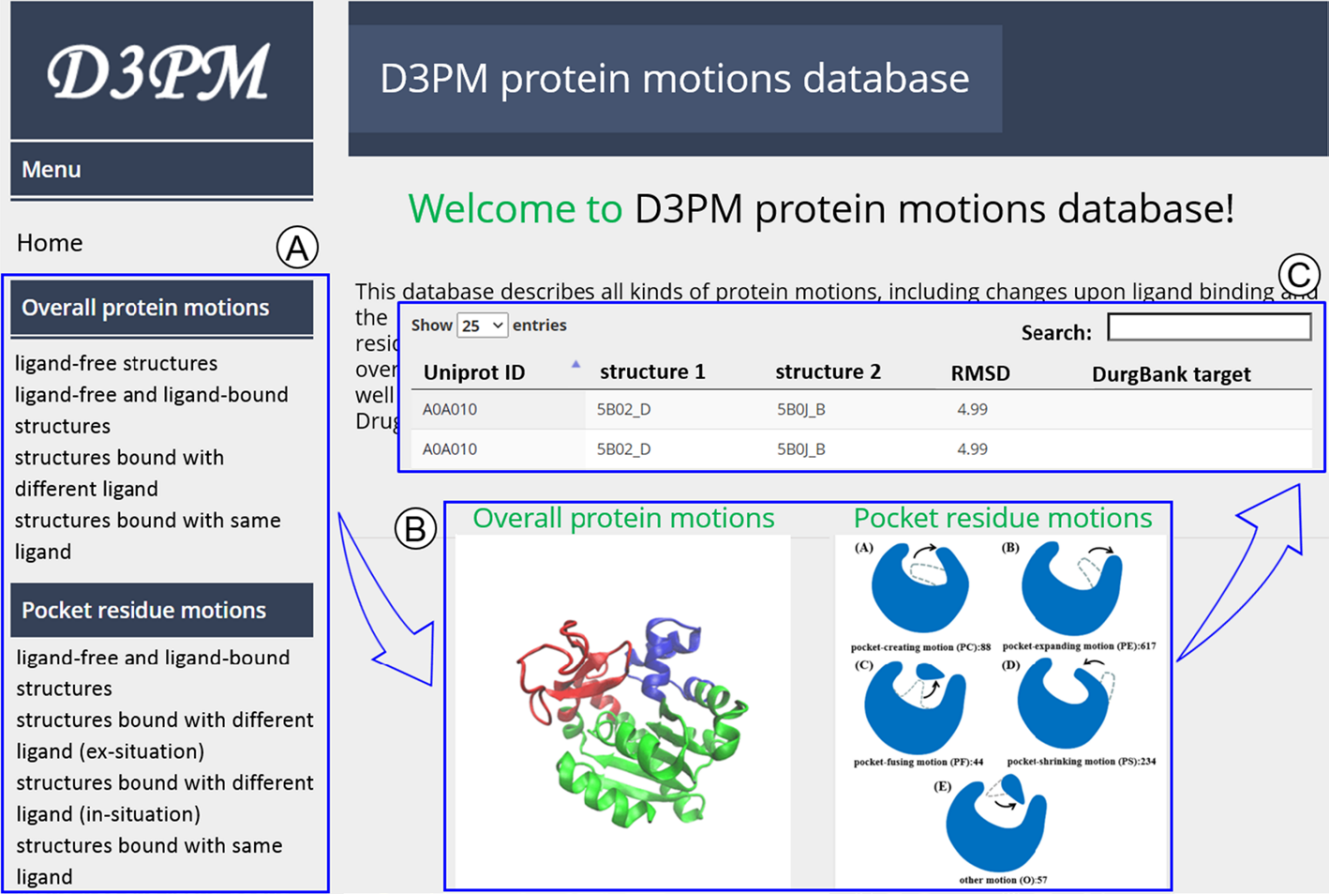
The web page of the D3PM database: **A** the overview of types of protein motions included in the D3PM, **B** diagrams for two main types of protein motions, viz*.* overall protein motions and pocket residue motions, **C** the detailed information of each protein motion

For easy application, we constructed a web server, which is accessible at www.d3pharma.com/D3PM/index.php (Fig. 2). The interface to the D3PM was designed to facilitate both detailed searching of protein motions and browsing of the whole database. In the website, users can navigate the protein motion list, and search the database by PDB ID, Uniprot ID, RMSD, residue and ligand name etc. Each entry includes detailed annotations such as PDB ID, Uniprot ID, overall RMSD, pocket RMSD etc. The D3PM has provided entries to download all the data. Three dimensional structures of pocket motions could been shown by JSmol software [43] after clicking the “structure” button. For the structural pairs, the first and second structures are highlighted in red and yellow, respectively. The user has the option to show the aligned structures in cartoon or sticks with the label of pocket residue’s name, and the option to download PDB files containing the aligned structures.

### Comparison of different types of protein motions

The D3PM provides sufficient samples to study protein motions caused by either the inherent flexibility of macromolecule or ligand binding. In the D3PM, 7,730,788 protein pairs are classified into four classes, viz. (a) pairs of apo structures, (b) apo-holo pairs, (c) pairs bound with different ligands, (d) pairs bound with the same ligand. By searching the database, we found 1970 proteins forming 3,990,497 protein pairs, among which each protein possesses all the 4 different motion types.

**Fig. 3:**
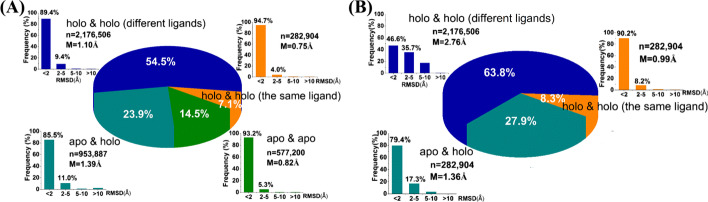
The frequency of four types of protein overall motions (**A**) and three types of pocket residue motions (**B**). The “apo” referred to as ligand-free protein, and the “holo” referred to as ligand-bound protein. The “n” refers to the total number of pairs, and the “M” refers to the mean value of RMSD

If a protein pair has overall RMSD that is smaller than 2.0 Å, it was regarded as motionless. In Fig. 3A, the pairs bound with the same ligand have a larger proportion of motionless pairs (94.7%) than that of the pairs of apo structures (93.2%), indicating the weaker ability of the ligand-bound proteins to undergo overall structural motions. The result can be rationalized with the fact that ligand somewhat stabilizes protein structure. However, it is noteworthy that there are nearly 5% of protein pairs bound with the same ligand that have RMSD that is greater than 2.0 Å, which is largely accomplished by flexible loops such as the active loop of kinases. The proportion of motionless for protein pairs bound with different ligands is 89.4%, which is 5.3% less than the pairs bound the same ligand. It indicated that the protein conformational adaptation induced by ligands is somewhat related to the structure of ligand. The apo-holo pairs have the smallest proportion of motionless pairs (85.5%), showing that the ligand binding causes the most significant protein motions. However, it is important to note that most proteins have no obvious overall structural changes upon ligand binding, because the proportions of motionless pairs for all the 4 types of motions are greater than 85%.

To explore pocket residue motions, the RMSD of pocket residues that around ligand by 5.0 Å was calculated (Fig. 3B). Similarly, the bound ligand reduces the flexibility of protein binding pocket, according to the largest proportion of RMSD smaller than 2.0 Å (90.2%) of the pairs bound with the same ligand. However, protein conformational change induced by ligands is much significant on binding pocket, the proportion of motionless pairs with different ligands (46.6%, Fig. 3B) is 42.8% less than that of overall structure (89.4%, Fig. 3A). In other words, more than half of pocket residues have significant structural changes upon ligand binding, implying the importance of the flexibility of pocket residues for virtual screening. In addition, the pairs bound with different ligands have the largest mean value of RMSD (2.76 Å). Therefore, all the results demonstrated that the ligand binding could cause protein conformational changes, especially in binding pocket, however, could also stabilize induced conformations.

### The amino acid preference of binding pocket

**Fig. 4:**
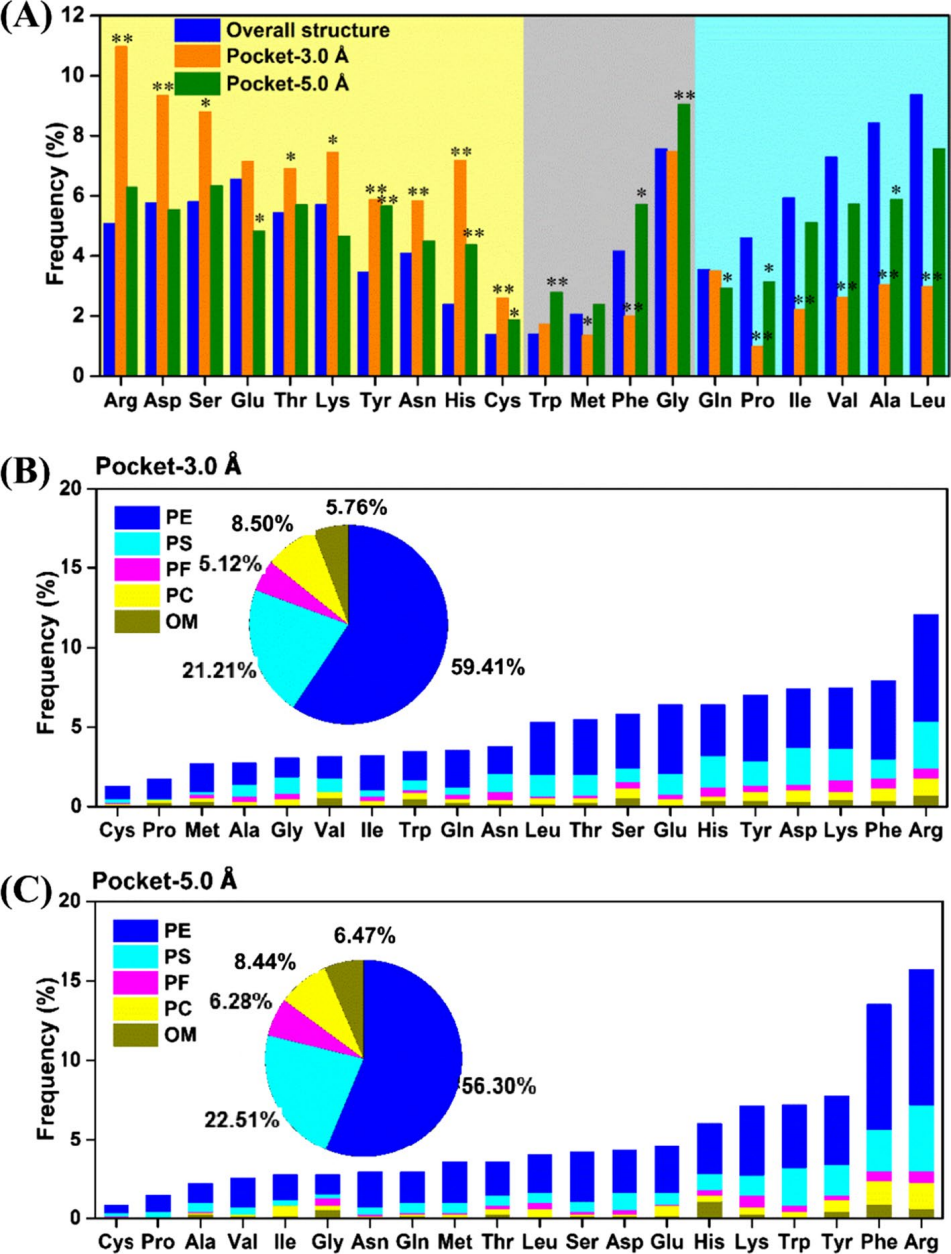
**A** Frequencies of 20 amino acid residues in overall protein structure (blue) or binding sites around ligand by 3.0 Å (orange) and 5.0 Å (green). The residues are grouped in yellow, gray and cyan blocks, according to the largest frequency belongs to pocket-3.0 Å, pocket-5.0 Å and overall structure, respectively. (*) the difference between the overall structure and binding site around ligand by 3.0 or 5.0 Å is statistically significant at the 5% level (*p* < 0.05). (**) the difference between the overall structure and binding site around ligand by 3.0 or 5.0 Å is statistically very significant at the 1% level (*p* < 0.01). Frequencies of 20 amino acid residues easy to move within binding pocket defined with the cutoff of 3.0 (**B**) or 5.0 Å (**C**)

Interactions with pocket residues are indispensable to the binding process of ligands, e.g., hydrogen bond, hydrophobic interaction and so on. In order to evaluate how amino acids that bind to ligands (pocket residues) differ from that of overall structure, the residues around ligands and the whole protein were analyzed. Usually, the definition of pocket residues is the ones with a minimum distance to ligand shorter than 5.0 Å [44]. With 178,778 protein–ligand complexes, the residue frequency of binding pocket was calculated by using different distance that around ligand from 2.0 to 6.0 Å. The mean unsigned error (MUE) of the frequencies of the 20 amino acids between binding pocket and overall structure was calculated (Additional file 1: Fig. S1). The distance of 3.0 Å has the largest difference of residue frequencies between binding pocket and overall structure. The larger the distance than 3.0 Å, the smaller the difference, indicating that the cutoff of 3.0 Å could best distinguish binding pocket from overall structure. Therefore, using the distances of pocket residues to ligand of 3.0 and 5.0 Å, we analyzed the frequencies of 20 amino acids for binding pocket and for overall structure, respectively. The Arg, Asp, Ser, Glu, Thr, Lys, Tyr, Asn, His and Cys in binding pocket around ligand by 3.0 Å significantly overweigh that in overall structure (Fig. 4A), indicating that they are more inclined to interact with ligands to form short-range interactions such as hydrogen bond and ionic bond. The frequencies of Gly, Phe, Met and Trp within 5.0 Å of ligands overweigh the corresponding ones in overall structure, indicating they are more inclined to interact with ligands to form long-range interactions. The 14 residues that are likely to interact with ligands to form short-range or long-range interactions, could be called “pocketphilic”. Other residues like Leu, Ala, Val, Ile, Pro and Gln have lower frequencies in binding pocket compared with overall structure with both the cutoff of 3.0 and 5.0 Å, which could be called “pocketphobic”.

To further explore the frequency of pocket residues that are easy to motion in responding to ligand binding, we analyzed the pocket residue motions in the D3PM. As shown in Fig. 4B & C, most motions are PE type with a frequency that is greater than 56%. The “pocketphilic” residues (Arg, Phe, Tyr, Lys, Glu, and Asp) are easier to move than “pocketphobic” residues. The Arg that has the longest side chain is the easiest to motion. However, it is not necessarily that the longer the side chain of residue is, the easier it is to move. For example, the motion frequency of Tyr is smaller than that of Phe. It is also interesting to note that basic residues (Arg and Lys) are easier to move than acidic residues (Asp and Glu) within ligand binding pockets.

### Case study: cross-docking reveals the importance of the pocket residue motions

The current strategy of virtual screening using a selected inhibitor bound conformation as receptor structure may miss putative ligands, due to protein conformational adaptations in ligand binding site. To evaluate how significant conformational adaptations are for molecular docking, the set of holo proteins bound with different ligands was used for cross-docking of ligand to a bound receptor structure crystallized in the presence of another ligand. The results (Additional file 1: Table S2) showed that the average docking score for ligands docked to its cocrystallized receptor is − 9.24 kcal/mol, however, the value is obvious smaller for ligands docked to other holo structures of the same protein, which is − 8.67 kcal/mol. In addition, 23% cross-docking cases have a difference of docking score greater than 1 kcal/mol. Taking the difference of 1 kcal/mol as a threshold, the enrichment factor is 1.94. As also shown in receiver operating characteristic (ROC) curves (Additional file 1: Fig. S2), the area under ROC curve (AUC) is 0.70. Therefore, the results showed that the flexibility of pocket residue need to be considered carefully during virtual screening.

## Conclusions

We developed the D3PM database to analyze all kinds of protein motions involving overall structures and binding pocket residues. In addition, we classified pocket residue motions into 5 types for studying different function mechanism of ligand binding. Currently, the information provided in the D3PM is in list form. The D3PM will be regularly updated to reflect new entries in the PDB database.

Using the D3PM, we firstly compared the ability of different factors that are related to protein conformational changes. The results showed that protein motions induced by ligands are significant in binding pocket according to 53.4% of protein pairs have pocket RMSD greater than 2.0 Å, but only less than 15% of protein pairs have obvious overall conformational adaptation. However, there are nearly 5% of protein pairs bound with the same ligand have overall RMSD greater than 2.0 Å. Although factors of both external perturbations like ligand binding and intrinsic flexibility of macromolecule have been studied here, there are still other factors like pH, temperatures and mutation that can impact protein motions, which is valuable for further study.

In addition, we analyzed the preferences of 20 amino acids in binding pocket. The results revealed some residues likely to interact with ligands by forming short-range or long-range interactions, which could be called “pocketphilic”. However, “pocketphobic” residues like Leu, Ala, Val, Glu, Ile, Pro and Gln have smaller frequencies in binding pocket compared with that in overall structure. The results could provide important information for pocket prediction.

## Supplementary Information

**Additional file 1.** The Additional file 1 contains Figures S1 and S2, Tables S1 and S2. **Figure S1** shows the mean unsigned error of the frequencies of the 20 amino acids between the pocket and overall structure. **Figure S2** shows the ROC curve for the cross docking case. **Table S1** shows the list of crystallographic additives. **Table S2** shows the detailed docking scores of the cross docking case.

## Data Availability

The database generated and analyzed is available at www.d3pharma.com/D3PM/index.php.
