# Supplementary material for: D3PM: a comprehensive database for protein motions ranging from residue to domain
Source: BMC Bioinformatics. 2022 Feb 14;23:70. doi: 10.1186/s12859-022-04595-0 (PMC8845362; doi:10.1186/s12859-022-04595-0)
Supplement: Supplementary file 1 — Additional file 1. The Additional file 1 contains Figures S1 and S2, Tables S1 and S2. Figure S1 shows the mean unsigned error of the frequencies of the 20 amino acids between the pocket and overall structure. Figure S2 shows the ROC curve for the cross docking case. Table S1 shows the list of crystallographic additives. Table S2 shows the detailed docking scores of the cross docking case. [file 12859_2022_4595_MOESM1_ESM.docx]

***Supporting Information***

**D3PM: A Comprehensive Database for Protein Motions Ranging from Residue to Domain**

Cheng Peng,^1,2,#^ Xinben Zhang, ^1,#^ Zhijian Xu,^*,1,2^ Zhaoqiang Chen,^1^ Yanqing Yang,^1,2^ Tingting Cai,^1^ Weiliang Zhu^*,1,2,3^

^1^ CAS Key Laboratory of Receptor Research; Drug Discovery and Design Center, Shanghai Institute of Materia Medica, Chinese Academy of Sciences, 555 Zuchongzhi Road, Shanghai, 201203, China

^2^University of Chinese Academy of Sciences, No.19A Yuquan Road, Beijing, 100049, China

^3^Open Studio for Druggability Research of Marine Natural Products, Pilot National Laboratory for Marine Science and Technology (Qingdao), 1 Wenhai Road, Aoshanwei, Jimo, Qingdao, 266237, PR China.

^#^These authors contributed equally to this work.

*Corresponding author

E-mail: [zjxu@simm.ac.cn](mailto:zjxu@simm.ac.cn)(ZX); wlzhu@simm.ac.cn(WZ)


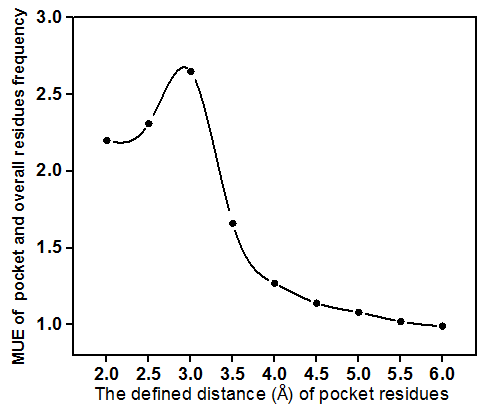


**Figure S1.** The mean unsigned error (MUE) of the frequencies of the 20 amino acids between the pocket and overall structure. The pocket residues were defined that around ligand by different distances, ranging from 2.0 Å to 6.0 Å.


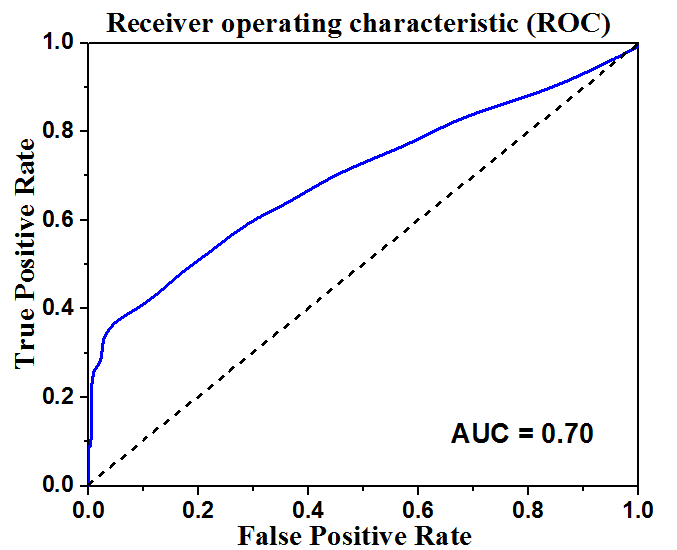


**Figure S2.** Fitted ROC curve for the cross-docking study. The area under ROC curve (AUC) is 0.70.

**Table S1.** **The list of crystallographic additives**

| Molecule | Structure_SMILES |
| --- | --- |
| GOL | OCC(O)CO |
| EDO | OCCO |
| PEG | OCCOCCO |
| MAN | OC[C@H]1O[C@H](O)[C@@H](O)[C@@H](O)[C@@H]1O |
| BMA | OC[C@H]1O[C@@H](O)[C@@H](O)[C@@H](O)[C@@H]1O |
| DMS | CS(C)=O |
| ACE | CC=O |
| MPD | C[C@H](O)CC(C)(C)O |
| MES | [O-]S(=O)(=O)CC[NH+]1CCOCC1 |
| TRS | [NH3+]C(CO)(CO)CO |
| PG4 | OCCOCCOCCOCCO |
| FUC | C[C@@H]1O[C@@H](O)[C@@H](O)[C@H](O)[C@@H]1O |
| GAL | OC[C@H]1O[C@@H](O)[C@H](O)[C@@H](O)[C@H]1O |
| EPE | OCCN1CCN(CCS(O)(=O)=O)CC1 |
| BME | OCCS |
| BGC | OC[C@H]1O[C@@H](O)[C@H](O)[C@@H](O)[C@@H]1O |
| IMD | c1c[nH+]c[nH]1 |
| GLC | OC[C@H]1O[C@H](O)[C@H](O)[C@@H](O)[C@@H]1O |
| 1PE | OCCOCCOCCOCCOCCO |
| IPA | CC(C)O |
| FLC | OC(CC([O-])=O)(CC([O-])=O)C([O-])=O |
| MRD | C[C@@H](O)CC(C)(C)O |
| BOG | CCCCCCCCO[C@@H]1O[C@H](CO)[C@@H](O)[C@H](O)[C@H]1O |
| TLA | O[C@H]([C@@H](O)C(O)=O)C(O)=O |
| P6G | OCCOCCOCCOCCOCCOCCO |
| SCN | [S-]C#N |
| CMO | [C-]#[O+] |
| CO3 | [O-]C([O-])=O |
| CAC | C[As](C)([O-])=O |
| XYP | O[C@@H]1CO[C@@H](O)[C@H](O)[C@H]1O |
| OLC | CCCCCCCC\C=C/CCCCCCCC(=O)OC[C@H](O)CO |
| EOH | CCO |
| LMT | CCCCCCCCCCCCO[C@@H]1O[C@H](CO)[C@@H](O[C@H]2O[C@H](CO)[C@@H](O)[C@H](O)[C@H]2O)[C@H](O)[C@H]1O |
| BEN | NC(=N)c1ccccc1 |
| LDA | CCCCCCCCCCCC[N+](C)(C)[O-] |
| HED | OCCSSCCO |
| C8E | CCCCCCCCOCCOCCOCCOCCO |
| CDL | CCCCCCCCCCCCCCCCCC(=O)OC[C@@H](CO[P@@]([O-])(=O)OC[C@H](O)CO[P@]([O-])(=O)OC[C@@H](COC(=O)CCCCCCCCCCCCCCCCC)OC(=O)CCCCCCCCCCCCCCCCC)OC(=O)CCCCCCCCCCCCCCCCC |
| 2PE | OCCOCCOCCOCCOCCOCCOCCOCCOCCO |
| HEZ | OCCCCCCO |
| DMU | CCCCCCCCCCO[C@@H]1O[C@H](CO)[C@@H](O[C@H]2O[C@H](CO)[C@@H](O)[C@H](O)[C@H]2O)[C@H](O)[C@H]1O |

**Table S2.** **Docking scores of cross-docking**

| Uniprot_ID | PDB_ID | Ligand | Docking_score | Protein_another | Docking_score | Difference |
| --- | --- | --- | --- | --- | --- | --- |
| A0A024B7W1 | 5MRK | SFG | -8.76 | 5KQS | -8.57 | 0.19 |
| A0A024B7W1 | 5KQS | SAM | -8.47 | 5MRK | -8.99 | 0.52 |
| A0A059WZ16 | 5F49 | MLC | -9.42 | 5F48 | -9.37 | 0.05 |
| A0A059WZ16 | 5F48 | COA | -8.77 | 5F49 | -9.18 | 0.41 |
| A0A0H2UPS5 | 2GZ3 | NAP | -10.24 | 2GZ2 | -10.77 | 0.53 |
| A0A0H2UPS5 | 2GZ2 | A2P | -8.75 | 2GZ3 | -9.25 | 0.50 |
| A0A0H2WY27 | 5TY2 | NFF | -7.81 | 5TW4 | -7.95 | 0.14 |
| A0A0H2WY27 | 5TW4 | AI8 | -9.66 | 5TY2 | -8.43 | 1.23 |
| A0A0H2Z9F5 | 5U8W | NAI | -10.94 | 5U8V | -10.93 | 0.01 |
| A0A0H2Z9F5 | 5U8V | NAD | -9.17 | 5U8W | -9.47 | 0.3 |
| A0A0H3LT39 | 3OZM | DXL | -6.23 | 3OP2 | -6.18 | 0.05 |
| A0A0H3LT39 | 3OP2 | AKG | -6.91 | 3OZM | -6.25 | 0.66 |
| A0A0J1HJU0 | 4MYX | 2F0 | -8.52 | 4MYA | -8.84 | 0.32 |
| A0A0J1HJU0 | 4MYA | 2EY | -9.13 | 4MYX | -8.77 | 0.36 |
| A0A0J9X1Y0 | 4D44 | JA3 | -8.47 | 4D43 | -8.05 | 0.42 |
| A0A0J9X1Y0 | 4D43 | 9W7 | -9.24 | 4D44 | -9.08 | 0.16 |
| A0A0R4I995 | 5CB4 | TIV | -10.78 | 5XI7 | -7.88 | 2.90 |
| A0A0R4I995 | 5XI7 | PO7 | -8.22 | 5CB4 | -8.63 | 0.41 |
| A0A0S7ASE9 | 5LCD | AMP | -8.52 | 5LDB | -8.02 | 0.5 |
| A0A0S7ASE9 | 5LDB | ADP | -9.06 | 5LCD | -9.37 | 0.31 |
| A0A0X1KGP2 | 3X1M | COA | -9.08 | 3X1J | -9.38 | 0.30 |
| A0A0X1KGP2 | 3X1J | ACO | -9.06 | 3X1M | -9.23 | 0.17 |
| A0A0X1KHF9 | 5FBT | 5WQ | -9.74 | 5FBU | -10.50 | 0.76 |
| A0A0X1KHF9 | 5FBU | 5WP | -11.30 | 5FBT | -9.77 | 1.53 |
| A0A117IMA6 | 5K5C | TRE | -7.37 | 5L3K | -8.20 | 0.83 |
| A0A117IMA6 | 5L3K | ADP | -8.73 | 5K5C | -8.37 | 0.36 |
| A0A1B0UHJ4 | 5KCQ | GST | -7.21 | 5KCL | -6.67 | 0.54 |
| A0A1B0UHJ4 | 5KCL | 6C7 | -6.50 | 5KCQ | -5.99 | 0.51 |
| A0R2B1 | 3ZHU | TD8 | -8.76 | 3ZHS | -7.62 | 1.14 |
| A0R2B1 | 3ZHS | TD6 | -7.57 | 3ZHU | -8.31 | 0.74 |
| A2QHE5 | 6EVD | BYN | -13.39 | 4ZA9 | -13.66 | 0.27 |
| A2QHE5 | 4ZA9 | 4MJ | -14.77 | 6EVD | -14.12 | 0.65 |
| A3FQ16 | 3IGO | ANP | -8.78 | 4Y5Q | -8.10 | 0.68 |
| A3FQ16 | 4Y5Q | AMP | -8.03 | 3IGO | -7.71 | 0.32 |
| A5H660 | 4BZ6 | SHH | -6.91 | 4CQF | -6.61 | 0.30 |
| A5H660 | 4CQF | 9Z8 | -5.25 | 4BZ6 | -5.03 | 0.22 |
| A5K1A2 | 2YND | 646 | -11.69 | 4CAF | -10.41 | 1.28 |
| A5K1A2 | 4CAF | 370 | -10.04 | 2YND | -8.88 | 1.16 |
| A5K1A2 | 5V0W | KFK | -11.38 | 2YND | -8.62 | 2.76 |
| A5K1A2 | 2YND | 646 | -10.19 | 5V0W | -10.37 | 0.18 |
| A5K4U6 | 5HN8 | HXK | -7.37 | 3RBM | -8.12 | 0.75 |
| A5K4U6 | 3RBM | B73 | -8.42 | 5HN8 | -7.58 | 0.84 |
| A5KE01 | 3EWD | MCF | -8.65 | 2PGR | -8.60 | 0.05 |
| A5KE01 | 2PGR | DCF | -8.91 | 3EWD | -7.74 | 1.17 |
| A6L2E5 | 5L7U | NGA | -6.59 | 5L7V | -6.12 | 0.47 |
| A6L2E5 | 5L7V | GNL | -7.40 | 5L7U | -6.94 | 0.46 |
| A6ZSR0 | 5ESG | 1YN | -11.73 | 4ZDY | -8.95 | 2.78 |
| A6ZSR0 | 5HS1 | VOR | -8.19 | 5ESG | -7.75 | 0.44 |
| A6ZSR0 | 4ZDY | 1YN | -11.87 | 5EAG | -10.88 | 0.99 |
| A6ZSR0 | 5EAG | 5LU | -7.00 | 5HS1 | -7.12 | 0.12 |
| A7Z2A9 | 5DM0 | 5D8 | -8.97 | 5DLY | -8.86 | 0.11 |
| A7Z2A9 | 5DLY | 5D7 | -7.39 | 5DM0 | -7.13 | 0.26 |
| A8C927 | 3P2K | SAM | -9.57 | 3P2E | -9.36 | 0.21 |
| A8C927 | 3P2E | SAH | -9.54 | 3P2K | -9.56 | 0.02 |
| A8DG50 | 4NWL | 2R9 | -9.25 | 4NWK | -9.90 | 0.65 |
| A8DG50 | 4NWK | 2R8 | -10.04 | 4NWL | -8.64 | 1.40 |
| A8DG50 | 3SU6 | SU3 | -12.28 | 3SU3 | -7.21 | 5.07 |
| A8DG50 | 3SV8 | SV6 | -6.57 | 3SU6 | -6.98 | 0.41 |
| A8DG50 | 3SU3 | SU3 | -12.57 | 3SUE | -7.57 | 5.00 |
| A8DG50 | 3SUE | SUE | -12.42 | 3SV8 | -11.04 | 1.38 |
| A8PWE4 | 2XGT | NSS | -12.16 | 2XTI | -10.74 | 1.42 |
| A8PWE4 | 2XTI | ATP | -11.05 | 2XGT | -11.36 | 0.31 |
| A9JQL9 | 2ZCQ | B65 | -9.69 | 3ACX | -9.76 | 0.07 |
| A9JQL9 | 3ACX | 673 | -9.77 | 2ZCQ | -8.88 | 0.89 |
| A9XG43 | 5C1U | GHV | -7.54 | 5DP7 | -7.50 | 0.04 |
| A9XG43 | 5DP7 | 5EC | -7.08 | 5C1U | -6.64 | 0.44 |
| B2HIZ6 | 4B55 | P18 | -6.05 | 2VFC | -5.54 | 0.51 |
| B2HIZ6 | 2VFC | COA | -7.13 | 4B55 | -7.98 | 0.85 |
| B2HVG8 | 3O83 | IXN | -10.19 | 3U16 | -9.60 | 0.59 |
| B2HVG8 | 3U16 | H89 | -10.10 | 3O83 | -9.51 | 0.59 |
| B3Q7C0 | 4GW9 | BLA | -11.65 | 4XTQ | -10.62 | 1.03 |
| B3Q7C0 | 4XTQ | BL8 | -10.71 | 4GW9 | -11.24 | 0.53 |
| B4Y381 | 4JEM | C5P | -8.86 | 4OHB | -8.54 | 0.32 |
| B4Y381 | 4OHB | 5HM | -8.66 | 4JEM | -9.54 | 0.88 |
| B5XYG3 | 4F7W | PN4 | -6.66 | 4GI7 | -6.62 | 0.04 |
| B5XYG3 | 4GI7 | 0JR | -7.81 | 4F7W | -7.16 | 0.65 |
| B6ZK72 | 4RZM | LSD | -9.55 | 3RGA | -9.87 | 0.32 |
| B6ZK72 | 3RGA | ILD | -10.87 | 4RZM | -8.42 | 2.45 |
| B8J605 | 4KAE | TGG | -8.74 | 4KDY | -8.35 | 0.39 |
| B8J605 | 4KDY | GSH | -6.60 | 4KAE | -7.35 | 0.75 |
| C0STY1 | 3W79 | ORI | -7.47 | 3W78 | -7.26 | 0.21 |
| C0STY1 | 3W78 | CBD | -9.23 | 3W79 | -10.02 | 0.79 |
| C1KIK8 | 5ESB | SU3 | -12.05 | 5ETX | -11.39 | 0.66 |
| C1KIK8 | 5ETX | 5RS | -9.28 | 5ESB | -8.74 | 0.54 |
| C3W5S0 | 4AWK | CI1 | -8.76 | 5WCS | -7.84 | 0.92 |
| C3W5S0 | 5WCS | 9KU | -9.62 | 4AWK | -9.51 | 0.11 |
| C4M4W4 | 4DVG | GSP | -10.88 | 3REF | -10.74 | 0.14 |
| C4M4W4 | 3REF | GDP | -9.97 | 4DVG | -6.26 | 3.71 |
| C7Q5P8 | 5BW4 | SAM | -8.73 | 5D1N | -9.42 | 0.69 |
| C7Q5P8 | 5D1N | SAH | -9.32 | 5BW4 | -8.77 | 0.55 |
| C8W8H7 | 4R1G | CXU | -8.17 | 4R3J | -8.53 | 0.36 |
| C8W8H7 | 4R3J | CFU | -7.11 | 4R1G | -7.03 | 0.08 |
| C9DRU9 | 4FWH | CIX | -8.22 | 4FWD | -8.15 | 0.07 |
| C9DRU9 | 4FWD | BO2 | -7.69 | 4FWH | -7.82 | 0.13 |
| D8KFM5 | 4N4D | XX8 | -9.90 | 4MHW | -7.65 | 2.25 |
| D8KFM5 | 4MHW | 0YN | -10.04 | 4N4D | -9.64 | 0.40 |
| D9J2T9 | 3RL9 | ADE | -5.59 | 4FXA | -5.78 | 0.19 |
| D9J2T9 | 4FXA | AAG | -6.12 | 3RL9 | -5.70 | 0.42 |
| E1BQ43 | 5KX5 | ADP | -9.99 | 5JCB | -9.08 | 0.91 |
| E1BQ43 | 5JCB | ACP | -9.23 | 5KX5 | -10.37 | 1.14 |
| E9QY26 | 4UYM | VOR | -8.14 | 4UYL | -7.89 | 0.25 |
| E9QY26 | 4UYL | VNI | -10.54 | 4UYM | -10.43 | 0.11 |
| G1C794 | 3UDX | IM2 | -6.35 | 3UE0 | -6.49 | 0.14 |
| G1C794 | 3UE0 | AZR | -7.42 | 3UDX | -7.42 | 0.00 |
| G1C794 | 3UE1 | UE1 | -9.71 | 3UDX | -9.52 | 0.19 |
| G1C794 | 3UDX | IM2 | -6.10 | 3UE1 | -6.43 | 0.33 |
| G4V7G8 | 3UQ9 | TBN | -9.28 | 3UQ6 | -9.24 | 0.04 |
| G4V7G8 | 3UQ6 | ADN | -8.87 | 3UQ9 | -8.86 | 0.01 |
| G7CGT2 | 5K44 | T6P | -8.03 | 5K41 | -8.07 | 0.04 |
| G7CGT2 | 5K41 | ADQ | -10.31 | 5K44 | -9.36 | 0.95 |
| G8G134 | 6C0M | 8LM | -8.95 | 5KV8 | -9.23 | 0.28 |
| G8G134 | 5KV8 | 6Y6 | -7.73 | 6C0M | -7.02 | 0.71 |
| G8NVB5 | 4XYB | NDP | -13.01 | 4XYE | -11.80 | 1.21 |
| G8NVB5 | 4XYE | NAD | -12.02 | 4XYB | -12.32 | 0.30 |
| I6L8L2 | 4B5D | SW4 | -8.00 | 4AFH | -7.21 | 0.79 |
| I6L8L2 | 4AFH | L0B | -8.78 | 4B5D | -8.09 | 0.69 |
| I6L8L8 | 4EPP | APR | -11.80 | 4EPQ | -9.93 | 1.87 |
| I6L8L8 | 4EPQ | 0RR | -10.20 | 4EPP | -6.62 | 3.58 |
| I6YFP0 | 4XU0 | 44N | -13.63 | 4XTU | -13.98 | 0.35 |
| I6YFP0 | 4XTU | 44J | -13.49 | 4XU0 | -12.72 | 0.77 |
| J9VFT1 | 5K8F | ATP | -11.15 | 5K8F | -9.70 | 1.45 |
| J9VFT1 | 5K8F | 6R9 | -10.31 | 5K8F | -10.26 | 0.05 |
| O00214 | 4HAN | NAD | -8.77 | 5T7I | -8.22 | 0.55 |
| O00214 | 5T7I | LAT | -6.30 | 4HAN | -5.65 | 0.65 |
| O00329 | 5M6U | 7KA | -8.25 | 5DXU | -7.96 | 0.29 |
| O00329 | 5DXU | 5H5 | -8.85 | 5M6U | -8.30 | 0.55 |
| O06543 | 2GD0 | MRS | -6.88 | 2YIM | -6.50 | 0.38 |
| O06543 | 2YIM | MC4 | -8.12 | 2GD0 | -8.36 | 0.24 |
| O06644 | 1T4C | COA | -10.08 | 1VGQ | -9.25 | 0.83 |
| O06644 | 1VGQ | CAO | -9.27 | 1T4C | -7.69 | 1.58 |
| O07347 | 2NG1 | GDP | -9.51 | 2C04 | -10.00 | 0.49 |
| O07347 | 2C04 | GCP | -10.56 | 2NG1 | -9.62 | 0.94 |
| O13833 | 4FHW | GTP | -8.75 | 4NKT | -9.33 | 0.58 |
| O13833 | 4NKT | 2KH | -8.26 | 4FHW | -9.35 | 1.09 |
| O14744 | 5EMM | 5QL | -11.08 | 5EMK | -10.81 | 0.27 |
| O14744 | 5EMK | 5QH | -11.61 | 5EMM | -10.90 | 0.71 |
| O14965 | 3UP2 | 0C8 | -9.79 | 3UO5 | -8.81 | 0.98 |
| O14965 | 4DHF | 0K6 | -9.47 | 3UP2 | -9.63 | 0.16 |
| O14965 | 3UO5 | 0BX | -9.15 | 3VAP | -8.98 | 0.17 |
| O14965 | 3VAP | 0FY | -10.48 | 4DHF | -9.28 | 1.20 |
| O14965 | 5DN3 | 5DN | -8.59 | 4ZTR | -8.56 | 0.03 |
| O14965 | 6HJJ | G7T | -11.38 | 5DN3 | -10.58 | 0.8 |
| O14965 | 5DR6 | 5E1 | -8.61 | 5DR6 | -7.63 | 0.98 |
| O14965 | 4ZTR | 4RJ | -11.87 | 6HJJ | -8.92 | 2.95 |
| O15264 | 4EYJ | N61 | -10.57 | 5EKN | -10.07 | 0.50 |
| O15264 | 5EKN | N58 | -10.00 | 4EYJ | -10.29 | 0.29 |
| O15826 | 2YB0 | DUR | -7.34 | 2CJE | -6.81 | 0.53 |
| O15826 | 2CJE | DUN | -9.60 | 2YB0 | -9.33 | 0.27 |
| O15826 | 2YAZ | UMP | -8.84 | 2YB0 | -8.96 | 0.12 |
| O15826 | 2YB0 | DUR | -7.65 | 2YAZ | -7.63 | 0.02 |
| O22476 | 4OH4 | ANP | -9.45 | 5LPB | -8.67 | 0.78 |
| O22476 | 5LPB | ADP | -7.28 | 4OH4 | -7.74 | 0.46 |
| O25511 | 2GN9 | UPG | -10.70 | 2GNA | -9.93 | 0.77 |
| O25511 | 2GNA | GDU | -10.07 | 2GN9 | -10.45 | 0.38 |
| O26232 | 3WK3 | O7E | -7.80 | 3NQE | -8.15 | 0.35 |
| O26232 | 3NQE | BMP | -9.40 | 3WK3 | -8.89 | 0.51 |
| O28344 | 4GVQ | N4M | -10.54 | 4GVS | -9.19 | 1.35 |
| O28344 | 4GVS | F4M | -8.37 | 4GVQ | -9.50 | 1.13 |
| O30297 | 1Z0U | NAP | -8.93 | 1Z0Z | -8.55 | 0.38 |
| O30297 | 1Z0Z | NAD | -9.27 | 1Z0U | -8.95 | 0.32 |
| O31801 | 4B0H | DUR | -6.69 | 2XCE | -6.12 | 0.57 |
| O31801 | 2XCE | DUP | -6.63 | 4B0H | -6.40 | 0.23 |
| O32080 | 2HMT | NAI | -10.09 | 4J91 | -9.60 | 0.49 |
| O32080 | 4J91 | ADP | -10.37 | 2HMT | -8.36 | 2.01 |
| O32727 | 1TID | ATP | -9.35 | 1TH8 | -9.51 | 0.16 |
| O32727 | 1TH8 | ADP | -9.24 | 1TID | -9.49 | 0.25 |
| O33877 | 4B0I | KBP | -4.75 | 4B0B | -4.73 | 0.02 |
| O33877 | 4B0B | 54F | -5.36 | 4B0I | -6.02 | 0.66 |
| O34453 | 4D3K | 12S | -11.02 | 4D3K | -10.50 | 0.52 |
| O34453 | 4UGR | 6E5 | -10.83 | 4D3K | -10.28 | 0.55 |
| O34453 | 4UGF | Q16 | -9.83 | 5G6F | -10.27 | 0.44 |
| O34453 | 4D3M | IVU | -11.28 | 4UGR | -11.36 | 0.08 |
| O34453 | 4D3K | 12S | -10.75 | 4D3M | -10.99 | 0.24 |
| O34453 | 5G6F | 2SN | -9.69 | 4UGF | -9.68 | 0.01 |
| O34757 | 3EHH | ADP | -10.34 | 5IUN | -10.33 | 0.01 |
| O34757 | 5IUN | ACP | -10.04 | 3EHH | -8.54 | 1.50 |
| O34919 | 4AO5 | UMP | -5.46 | 2Y1T | -6.25 | 0.79 |
| O34919 | 2Y1T | DUD | -5.90 | 4AO5 | -5.79 | 0.11 |
| O35904 | 5IS5 | 6CY | -10.87 | 5I6U | -10.00 | 0.87 |
| O35904 | 6FTN | E78 | -10.34 | 5IS5 | -8.17 | 2.17 |
| O35904 | 5I6U | 68R | -10.76 | 6FTN | -8.85 | 1.91 |
| O35904 | 6FTN | E78 | -10.34 | 6FTN | -7.78 | 2.56 |
| O35963 | 1Z06 | GNP | -12.82 | 2G77 | -12.37 | 0.45 |
| O35963 | 2G77 | GDP | -10.73 | 1Z06 | -10.84 | 0.11 |
| O43173 | 5BO6 | CDP | -10.61 | 5CXY | -10.17 | 0.44 |
| O43173 | 5CXY | 55T | -11.66 | 5BO6 | -11.15 | 0.51 |
| O49686 | 5UR6 | 8KM | -8.69 | 3WG8 | -8.79 | 0.10 |
| O49686 | 5YGV | 8V6 | -9.23 | 5UR6 | -8.77 | 0.46 |
| O49686 | 3WG8 | 6AS | -8.05 | 5YGV | -8.27 | 0.22 |
| O49686 | 5YGV | 8V6 | -9.23 | 5YGV | -10.32 | 1.09 |
| O54438 | 4BO3 | U98 | -7.01 | 4BNX | -6.56 | 0.45 |
| O54438 | 4BNX | O74 | -7.50 | 4BO3 | -7.44 | 0.06 |
| O57883 | 2DTH | ADP | -7.08 | 2ZGW | -6.87 | 0.21 |
| O57883 | 2ZGW | ADN | -6.95 | 2DTH | -7.22 | 0.27 |
| O59521 | 5H7K | GDP | -10.27 | 5H7J | -9.21 | 1.06 |
| O59521 | 5H7J | GCP | -8.62 | 5H7K | -8.64 | 0.02 |
| O60218 | 4I5X | FLF | -9.89 | 5LIU | -9.33 | 0.56 |
| O60218 | 5LIU | 388 | -8.88 | 4I5X | -9.33 | 0.45 |
| O60674 | 4JI9 | 1M3 | -9.78 | 4GFM | -9.65 | 0.13 |
| O60674 | 5L3A | 6DP | -6.46 | 4JI9 | -6.77 | 0.31 |
| O60674 | 5CF8 | 50V | -9.27 | 5CF8 | -8.63 | 0.64 |
| O60674 | 4GFM | 0X2 | -7.95 | 5L3A | -7.42 | 0.53 |
| O60674 | 5WIN | SKE | -9.61 | 6M9H | -10.71 | 1.1 |
| O60674 | 6M9H | J9D | -8.76 | 5WIN | -8.29 | 0.47 |
| O60885 | 2YEM | WSH | -10.44 | 5UEU | -8.67 | 1.77 |
| O60885 | 5UEU | 0S6 | -8.16 | 2YEM | -8.60 | 0.44 |
| O67135 | 1C3R | TSN | -8.57 | 1C3S | -8.55 | 0.02 |
| O67135 | 1C3S | SHH | -9.31 | 1C3R | -8.33 | 0.98 |
| O67575 | 5TV8 | APC | -9.88 | 5TVA | -9.48 | 0.4 |
| O67575 | 5TVA | AMP | -9.86 | 5TV8 | -9.14 | 0.72 |
| O67648 | 5DRP | 5EP | -9.62 | 3P3C | -10.14 | 0.52 |
| O67648 | 3P3C | 3P3 | -10.49 | 5DRP | -10.14 | 0.35 |
| O70370 | 4BSQ | QQV | -7.64 | 4MZS | -7.94 | 0.3 |
| O70370 | 4BQV | 8PW | -7.89 | 4BQV | -7.33 | 0.56 |
| O70370 | 4BS6 | JG7 | -7.79 | 4BS6 | -7.40 | 0.39 |
| O70370 | 4MZS | 2EV | -8.11 | 4BSQ | -7.85 | 0.26 |
| O73948 | 3NTU | ANP | -8.51 | 2FPK | -8.72 | 0.21 |
| O73948 | 2FPK | ADP | -8.42 | 3NTU | -8.28 | 0.14 |
| O74036 | 4D6P | ANP | -8.29 | 4UQO | -8.63 | 0.34 |
| O74036 | 4UQO | ADP | -7.31 | 4D6P | -8.12 | 0.81 |
| O75460 | 4YZC | STU | -13.22 | 4Z7H | -6.54 | 6.68 |
| O75460 | 4Z7H | 4L5 | -9.03 | 4YZC | -8.17 | 0.86 |
| O75469 | 6BNS | XGH | -10.78 | 4NY9 | -10.67 | 0.11 |
| O75469 | 4NY9 | 2Q4 | -9.60 | 6BNS | -8.96 | 0.64 |
| O76290 | 4CMK | FQW | -9.42 | 3BMO | -9.13 | 0.29 |
| O76290 | 3BMO | AX4 | -6.97 | 4CMK | -7.04 | 0.07 |
| O80992 | 3NJ1 | PYV | -8.91 | 3KB3 | -9.02 | 0.11 |
| O80992 | 3KB3 | A8S | -8.68 | 3NJ1 | -7.84 | 0.84 |
| O88703 | 5KHJ | 6SY | -11.39 | 5KHH | -10.21 | 1.18 |
| O88703 | 5KHH | 6SW | -11.14 | 5KHJ | -10.55 | 0.59 |
| O92972 | 4JU1 | 1NZ | -11.15 | 4JTZ | -11.86 | 0.71 |
| O92972 | 4JTZ | 1NW | -11.61 | 4JU1 | -10.92 | 0.69 |
| O95271 | 4N4V | 2GY | -12.69 | 4MSK | -8.67 | 4.02 |
| O95271 | 4MSK | 2C8 | -14.76 | 4N4V | -12.82 | 1.94 |
| O95819 | 4U42 | 3C8 | -10.57 | 4OBQ | -8.88 | 1.69 |
| O95819 | 4OBQ | 2QT | -10.63 | 4U42 | -10.49 | 0.14 |
| O96013 | 5VEF | M77 | -8.25 | 5VEE | -7.04 | 1.21 |
| O96013 | 5VEE | 981 | -8.52 | 5VEF | -8.62 | 0.1 |
| O96935 | 4K5P | 1OS | -8.79 | 4K5L | -8.01 | 0.78 |
| O96935 | 4K5L | 19N | -7.05 | 4K5P | -7.19 | 0.14 |
| P00157 | 2FYU | FDN | -11.99 | 1SQB | -8.70 | 3.29 |
| P00157 | 1SQB | AZO | -8.74 | 2FYU | -8.42 | 0.32 |
| P00178 | 4H1N | CGE | -7.93 | 3UAS | -7.61 | 0.32 |
| P00178 | 3UAS | 0BV | -8.99 | 4H1N | -8.49 | 0.50 |
| P00179 | 1N6B | DMZ | -7.65 | 1NR6 | -8.32 | 0.67 |
| P00179 | 1NR6 | DIF | -7.46 | 1N6B | -7.27 | 0.19 |
| P00349 | 1PGO | NDP | -8.75 | 1PGN | -9.07 | 0.32 |
| P00349 | 1PGN | NBP | -9.33 | 1PGO | -9.69 | 0.36 |
| P00374 | 3S7A | 684 | -10.53 | 4KAK | -10.45 | 0.08 |
| P00374 | 4KAK | 06U | -10.51 | 3S7A | -9.76 | 0.75 |
| P00374 | 3FS6 | DH1 | -8.38 | 3NXT | -7.69 | 0.69 |
| P00374 | 3NXT | D2E | -8.84 | 3FS6 | -9.47 | 0.63 |
| P00438 | 1PDH | FAS | -13.17 | 1PBF | -12.14 | 1.03 |
| P00438 | 1PBF | FAD | -13.42 | 1PDH | -12.71 | 0.71 |
| P00439 | 4PAH | LNR | -7.15 | 4ANP | -7.33 | 0.18 |
| P00439 | 4ANP | 3QI | -7.66 | 4PAH | -8.19 | 0.53 |
| P00519 | 2HYY | STI | -12.91 | 2HZ0 | -9.92 | 2.99 |
| P00519 | 2HZ0 | GIN | -11.82 | 2HYY | -10.80 | 1.02 |
| P00520 | 3KFA | B91 | -14.15 | 3IK3 | -12.98 | 1.17 |
| P00520 | 3IK3 | 0LI | -13.81 | 3KFA | -10.58 | 3.23 |
| P00523 | 5D10 | 563 | -9.84 | 4O2P | -9.06 | 0.78 |
| P00523 | 4O2P | 11V | -9.29 | 5D10 | -7.66 | 1.63 |
| P00636 | 1FBF | AHM | -7.16 | 1FBE | -7.72 | 0.56 |
| P00636 | 1FBE | AHG | -7.41 | 1FBF | -7.21 | 0.2 |
| P00653 | 1RDS | GPC | -10.17 | 1RMS | -8.06 | 2.11 |
| P00653 | 1RMS | 3GP | -8.21 | 1RDS | -7.69 | 0.52 |
| P00720 | 5CGD | 51E | -11.78 | 4OO9 | -7.89 | 3.89 |
| P00720 | 4OO9 | 2U8 | -12.10 | 5CGD | -6.40 | 5.7 |
| P00734 | 4BAQ | M4Z | -10.32 | 3TU7 | -8.83 | 1.49 |
| P00734 | 1T4U | 81A | -9.32 | 1T4U | -8.89 | 0.43 |
| P00734 | 1YPK | CCR | -10.02 | 1YPK | -8.69 | 1.33 |
| P00734 | 3TU7 | 0BM | -9.81 | 4BAQ | -9.29 | 0.52 |
| P00742 | 3HPT | YET | -10.01 | 2RA0 | -9.39 | 0.62 |
| P00742 | 2RA0 | JNJ | -11.51 | 3HPT | -9.89 | 1.62 |
| P00760 | 1Y5B | TL3 | -8.53 | 1V2N | -8.69 | 0.16 |
| P00760 | 1V2N | BBA | -10.30 | 1Y5B | -8.61 | 1.69 |
| P00772 | 1ELE | 0QN | -7.42 | 1BMA | -7.38 | 0.04 |
| P00772 | 1BMA | 0QH | -7.99 | 1ELE | -7.54 | 0.45 |
| P00772 | 1E34 | TPX | -5.83 | 2EST | -5.44 | 0.39 |
| P00772 | 2EST | 2Z5 | -7.59 | 1E34 | -7.71 | 0.12 |
| P00797 | 1BIL | 0IU | -10.36 | 1HRN | -9.90 | 0.46 |
| P00797 | 1HRN | 03D | -8.89 | 1BIL | -9.53 | 0.64 |
| P00953 | 1I6K | TYM | -12.44 | 5DK4 | -12.28 | 0.16 |
| P00953 | 5DK4 | 5BX | -8.15 | 1I6K | -7.92 | 0.23 |
| P01837 | 2AJX | TGN | -5.68 | 2AJY | -5.12 | 0.56 |
| P01837 | 2AJY | ECG | -4.21 | 2AJX | -4.45 | 0.24 |
| P02638 | 4FQO | AZ3 | -5.14 | 5DKR | -5.45 | 0.31 |
| P02638 | 5DKR | 5CZ | -7.48 | 4FQO | -7.02 | 0.46 |
| P02701 | 5MYQ | HBF | -9.54 | 5IRU | -9.04 | 0.5 |
| P02701 | 5IRU | B9P | -10.72 | 5MYQ | -9.75 | 0.97 |
| P02753 | 5NU7 | RTL | -9.90 | 3FMZ | -9.03 | 0.87 |
| P02753 | 3FMZ | 2T1 | -11.59 | 5NU7 | -9.35 | 2.24 |
| P02768 | 4L9Q | 9TP | -12.15 | 2XW1 | -10.42 | 1.73 |
| P02768 | 3LU8 | IQX | -9.27 | 4L9Q | -9.61 | 0.34 |
| P02768 | 2XW1 | 9NV | -8.40 | 5YOQ | -6.18 | 2.22 |
| P02768 | 5YOQ | CLT | -6.85 | 3LU8 | -6.49 | 0.36 |
| P02879 | 4HV3 | 19L | -9.23 | 4HV7 | -10.07 | 0.84 |
| P02879 | 4HV7 | 19J | -8.09 | 4HV3 | -7.59 | 0.5 |
| P03195 | 2WE1 | UMP | -8.50 | 2WE3 | -7.73 | 0.77 |
| P03195 | 2WE3 | DUT | -8.19 | 2WE1 | -7.95 | 0.24 |
| P03303 | 1VRH | SD8 | -10.19 | 1R09 | -9.09 | 1.10 |
| P03303 | 1R09 | JEN | -9.56 | 1VRH | -9.04 | 0.52 |
| P03366 | 4RW9 | 3X6 | -11.15 | 4O4G | -9.59 | 1.56 |
| P03366 | 4O4G | 2RT | -10.59 | 4RW9 | -10.74 | 0.15 |
| P03366 | 1NPV | L27 | -8.15 | 1HVK | -7.32 | 0.83 |
| P03366 | 1HVK | A79 | -6.80 | 1NPV | -7.44 | 0.64 |
| P03372 | 4IWC | 1GV | -10.64 | 4IWF | -6.50 | 4.14 |
| P03372 | 4IWF | 15Q | -9.15 | 4IWC | -9.14 | 0.01 |
| P03819 | 3EYW | NAD | -10.41 | 3L9W | -9.81 | 0.60 |
| P03819 | 3L9W | AMP | -9.98 | 3EYW | -8.16 | 1.82 |
| P03951 | 4CRC | OTM | -10.22 | 1ZPB | -5.85 | 4.37 |
| P03951 | 1ZPB | 995 | -7.81 | 4CRC | -7.11 | 0.70 |
| P04035 | 1DQ9 | HMG | -6.63 | 3CD5 | -7.35 | 0.72 |
| P04035 | 3CD5 | 7HI | -8.50 | 1DQ9 | -8.41 | 0.09 |
| P04058 | 2CEK | N8T | -12.54 | 1ZGB | -12.52 | 0.02 |
| P04058 | 1ZGB | A1E | -11.24 | 2CEK | -11.61 | 0.37 |
| P04150 | 6EL7 | B9T | -9.70 | 5UC3 | -5.97 | 3.73 |
| P04150 | 5UC3 | 486 | -4.46 | 6EL7 | -0.07 | 4.39 |
| P04181 | 2OAT | PFM | -7.63 | 5VWO | -6.78 | 0.85 |
| P04181 | 5VWO | 9QJ | -7.23 | 2OAT | -8.07 | 0.84 |
| P04585 | 1JLB | NVP | -10.18 | 3DLG | -10.20 | 0.02 |
| P04585 | 3DLG | GWE | -10.24 | 1JLB | -10.21 | 0.03 |
| P04695 | 1TND | GSP | -14.13 | 1TAG | -12.72 | 1.41 |
| P04695 | 1TAG | GDP | -13.12 | 1TND | -13.18 | 0.06 |
| P04825 | 2DQM | BES | -9.91 | 5MFR | -8.41 | 1.50 |
| P04825 | 5MFR | 7MK | -8.87 | 2DQM | -8.31 | 0.56 |
| P04925 | 4MA8 | Z80 | -5.79 | 4MA7 | -5.94 | 0.15 |
| P04925 | 4MA7 | P2Z | -5.86 | 4MA8 | -5.70 | 0.16 |
| P05326 | 3ZKU | HCV | -7.32 | 2JB4 | -7.23 | 0.09 |
| P05326 | 2JB4 | A14 | -7.96 | 3ZKU | -7.38 | 0.58 |
| P05798 | 2SAR | 3GP | -7.23 | 1GMP | -7.71 | 0.48 |
| P05798 | 1GMP | 2GP | -8.41 | 2SAR | -7.31 | 1.10 |
| P06401 | 3ZRA | ORB | -9.41 | 2W8Y | -9.19 | 0.22 |
| P06401 | 2W8Y | 486 | -6.54 | 3ZRA | 8.53 | 15.07 |
| P06401 | 3KBA | WOW | -10.25 | 3ZRA | -8.52 | 1.73 |
| P06401 | 3ZRA | ORB | -9.19 | 3KBA | -8.85 | 0.34 |
| P06730 | 2V8W | MGO | -9.36 | 1IPB | -9.46 | 0.10 |
| P06730 | 1IPB | GTA | -8.97 | 2V8W | -9.19 | 0.22 |
| P06737 | 3CEM | AVD | -10.51 | 3DDS | -8.35 | 2.16 |
| P06737 | 3DDS | 26B | -9.14 | 3CEM | -8.05 | 1.09 |
| P06745 | 1U0F | G6P | -8.91 | 1U0G | -8.91 | 0.00 |
| P06745 | 1U0G | E4P | -6.46 | 1U0F | -6.20 | 0.26 |
| P07097 | 1QFL | COA | -7.98 | 1OU6 | -10.27 | 2.29 |
| P07097 | 1OU6 | 168 | -7.05 | 1QFL | -6.13 | 0.92 |
| P07097 | 2VU2 | PN5 | -5.59 | 2WKV | -6.06 | 0.47 |
| P07097 | 2WKV | COA | -7.41 | 2VU2 | -7.84 | 0.43 |
| P07251 | 3ZIA | ATP | -9.95 | 3ZIA | -9.37 | 0.58 |
| P07251 | 3ZIA | ADP | -9.17 | 3ZIA | -8.19 | 0.98 |
| P07332 | 3BKB | STU | -13.60 | 4E93 | -9.12 | 4.48 |
| P07332 | 4E93 | GUI | -9.67 | 3BKB | -9.60 | 0.07 |
| P07333 | 3KRL | KRL | -8.80 | 2I1M | -9.78 | 0.98 |
| P07333 | 2I1M | 5CN | -9.35 | 3KRL | -8.27 | 1.08 |
| P07711 | 4AXM | V65 | -7.87 | 3BC3 | -7.93 | 0.06 |
| P07711 | 3BC3 | OPT | -7.13 | 4AXM | -6.91 | 0.22 |
| P07788 | 4Q8B | SXX | -5.22 | 1OF0 | -4.98 | 0.24 |
| P07788 | 1OF0 | EBS | -7.66 | 4Q8B | -8.40 | 0.74 |
| P07814 | 4HVC | ANP | -11.46 | 5V58 | -11.17 | 0.29 |
| P07814 | 5V58 | 8X1 | -12.43 | 4HVC | -11.52 | 0.91 |
| P07858 | 6AY2 | C1G | -6.88 | 1GMY | -7.30 | 0.42 |
| P07858 | 1GMY | APD | -5.78 | 6AY2 | -5.71 | 0.07 |
| P07900 | 5J20 | 6FJ | -10.56 | 4AWQ | -10.16 | 0.40 |
| P07900 | 4CWF | H05 | -9.27 | 5J20 | -8.41 | 0.86 |
| P07900 | 4AWQ | 592 | -11.88 | 5J6L | -7.60 | 4.28 |
| P07900 | 5J6L | 6GC | -10.64 | 4CWF | -9.79 | 0.85 |
| P07949 | 2IVU | ZD6 | -9.36 | 4CKJ | -8.09 | 1.27 |
| P07949 | 4CKJ | ADN | -8.19 | 2IVU | -7.68 | 0.51 |
| P07986 | 3CUI | X4S | -9.72 | 3CUF | -9.85 | 0.13 |
| P07986 | 3CUF | 9MR | -7.40 | 3CUI | -7.69 | 0.29 |
| P08037 | 1TW5 | UDH | -9.84 | 1YRO | -9.89 | 0.05 |
| P08037 | 1YRO | GDU | -12.14 | 1TW5 | -11.14 | 1.00 |
| P08049 | 5V48 | TIO | -7.10 | 4ZR5 | -6.87 | 0.23 |
| P08049 | 4ZR5 | RDF | -10.82 | 5V48 | -7.80 | 3.02 |
| P08191 | 5L4W | 6KH | -7.49 | 4X50 | -6.76 | 0.73 |
| P08191 | 4X50 | 3X8 | -9.28 | 5L4W | -9.21 | 0.07 |
| P08246 | 3Q77 | 2HY | -8.77 | 1H1B | -7.14 | 1.63 |
| P08246 | 1H1B | 151 | -6.92 | 3Q77 | -6.56 | 0.36 |
| P08473 | 2YB9 | HA0 | -8.66 | 1R1H | -9.19 | 0.53 |
| P08473 | 1R1H | BIR | -9.20 | 2YB9 | -9.21 | 0.01 |
| P08473 | 4CTH | RDF | -10.36 | 1R1J | -9.01 | 1.35 |
| P08473 | 1R1J | OIR | -8.31 | 4CTH | -8.27 | 0.04 |
| P08519 | 4BV7 | BV7 | -4.85 | 4BV5 | -4.78 | 0.07 |
| P08519 | 4BV5 | 5C3 | -6.53 | 4BV7 | -7.43 | 0.90 |
| P08581 | 3C1X | CKK | -12.51 | 5DG5 | -10.55 | 1.96 |
| P08581 | 5DG5 | 5B4 | -12.62 | 3C1X | -9.92 | 2.70 |
| P08659 | 3IES | M24 | -13.23 | 5GYZ | -12.78 | 0.45 |
| P08659 | 5GYZ | 7BV | -10.78 | 3IES | -10.68 | 0.10 |
| P08684 | 4NY4 | 2QH | -11.73 | 3UA1 | -12.01 | 0.28 |
| P08684 | 3UA1 | 08Y | -13.87 | 4NY4 | -12.05 | 1.82 |
| P08709 | 4ZXY | 4T1 | -11.02 | 4Z6A | -11.36 | 0.34 |
| P08709 | 4Z6A | 0Z6 | -7.77 | 4ZXY | -7.73 | 0.04 |
| P09147 | 1XEL | UPG | -11.16 | 1NAI | -10.77 | 0.39 |
| P09147 | 1NAI | UDP | -8.90 | 1XEL | -9.64 | 0.74 |
| P09148 | 1HXP | UDP | -8.17 | 1HXP | -8.16 | 0.01 |
| P09148 | 1HXP | U5P | -7.32 | 1HXP | -7.30 | 0.02 |
| P09237 | 2Y6D | TQJ | -8.72 | 2Y6C | -7.46 | 1.26 |
| P09237 | 2Y6C | TQI | -8.39 | 2Y6D | -9.52 | 1.13 |
| P09467 | 3KBZ | 2T4 | -7.78 | 4MJO | -7.20 | 0.58 |
| P09467 | 4MJO | 2C1 | -7.99 | 3KBZ | -7.44 | 0.55 |
| P09838 | 4I2I | AP5 | -9.53 | 4IQT | -8.48 | 1.05 |
| P09838 | 4IQT | 1FO | -7.34 | 4I2I | -7.82 | 0.48 |
| P09874 | 1WOK | CNQ | -9.24 | 3L3M | -8.89 | 0.35 |
| P09874 | 1UK1 | FRQ | -11.22 | 1WOK | -9.57 | 1.65 |
| P09874 | 3L3M | A92 | -10.33 | 1UK0 | -10.34 | 0.01 |
| P09874 | 1UK0 | FRM | -11.27 | 1UK1 | -11.11 | 0.16 |
| P0A0N3 | 3BT9 | DEQ | -9.77 | 3BTI | -9.68 | 0.09 |
| P0A0N3 | 3BTI | BER | -10.30 | 3BT9 | -10.68 | 0.38 |
| P0A2K1 | 1TTQ | PLP | -9.34 | 2J9X | -9.19 | 0.15 |
| P0A2K1 | 2J9X | P1T | -10.98 | 1TTQ | -7.96 | 3.02 |
| P0A3R9 | 2CBQ | TH2 | -8.81 | 1NCO | -8.73 | 0.08 |
| P0A3R9 | 1NCO | CHR | -11.75 | 2CBQ | -8.52 | 3.23 |
| P0A3Y5 | 3TM0 | ANP | -9.22 | 1J7L | -9.81 | 0.59 |
| P0A3Y5 | 1J7L | ADP | -10.42 | 3TM0 | -9.30 | 1.12 |
| P0A6I6 | 6CCQ | EX7 | -9.05 | 1B6T | -8.39 | 0.66 |
| P0A6I6 | 1B6T | COD | -9.90 | 6CCQ | -7.66 | 2.24 |
| P0A6K3 | 1BSJ | MLN | -7.66 | 3K6L | -8.48 | 0.82 |
| P0A6K3 | 3K6L | 2BB | -8.17 | 1BSJ | -7.55 | 0.62 |
| P0A6R0 | 5BNR | 4VL | -9.51 | 5BNM | -8.64 | 0.87 |
| P0A6R0 | 1HNJ | MLC | -7.41 | 5BNR | -8.17 | 0.76 |
| P0A6R0 | 5BNM | 4VK | -9.07 | 5BNR | -9.72 | 0.65 |
| P0A6R0 | 5BNR | 4VL | -9.51 | 1HNJ | -8.91 | 0.60 |
| P0A786 | 2FZK | EOZ | -9.16 | 2FZG | -9.65 | 0.49 |
| P0A786 | 2FZG | EOB | -8.33 | 2FZK | -8.07 | 0.26 |
| P0A794 | 1HO4 | PXP | -8.05 | 1IXO | -6.93 | 1.12 |
| P0A794 | 1IXO | G3P | -5.80 | 1HO4 | -6.31 | 0.51 |
| P0A7F3 | 4FYY | CTP | -7.30 | 4AT1 | -7.42 | 0.12 |
| P0A7F3 | 4AT1 | ATP | -7.65 | 4FYY | -7.54 | 0.11 |
| P0A7G6 | 1XMS | ANP | -9.91 | 1XMV | -10.21 | 0.30 |
| P0A7G6 | 1XMV | ADP | -9.19 | 1XMS | -9.33 | 0.14 |
| P0A884 | 1JG0 | DDT | -10.43 | 1DDU | -10.47 | 0.04 |
| P0A884 | 1DDU | CB3 | -9.65 | 1JG0 | -7.85 | 1.80 |
| P0A8M3 | 1EVL | TSB | -11.87 | 4HWR | -10.79 | 1.08 |
| P0A8M3 | 4HWR | 1B2 | -10.94 | 1EVL | -11.52 | 0.58 |
| P0A988 | 4N95 | 2HQ | -5.52 | 4MJR | -5.33 | 0.19 |
| P0A988 | 4MJR | 0LA | -6.73 | 4N95 | -6.50 | 0.23 |
| P0A9J6 | 1RK2 | ADP | -9.86 | 1GQT | -9.35 | 0.51 |
| P0A9J6 | 1GQT | ACP | -9.66 | 1RK2 | -10.27 | 0.61 |
| P0A9M2 | 5KNV | 6W9 | -8.14 | 5KNS | -8.23 | 0.09 |
| P0A9M2 | 5KNS | 3L7 | -7.71 | 5KNV | -7.26 | 0.45 |
| P0ABE7 | 5N2R | 8JN | -8.67 | 5IUB | -9.05 | 0.38 |
| P0ABE7 | 5IUB | 6DV | -9.51 | 5N2R | -10.27 | 0.76 |
| P0AC14 | 5U10 | PT1 | -9.76 | 5U0Z | -9.11 | 0.65 |
| P0AC14 | 5U0Z | 7PM | -8.71 | 5U10 | -8.98 | 0.27 |
| P0ACT4 | 4ABZ | T1C | -10.04 | 2O7O | -8.62 | 1.42 |
| P0ACT4 | 2O7O | DXT | -10.98 | 4ABZ | -10.31 | 0.67 |
| P0AEB2 | 3MZD | CXV | -7.62 | 1Z6F | -8.24 | 0.62 |
| P0AEB2 | 1Z6F | BO9 | -7.66 | 3MZD | -8.28 | 0.62 |
| P0AEB2 | 5J8X | OK3 | -7.13 | 3MZF | -7.19 | 0.06 |
| P0AEB2 | 3MZF | IM2 | -6.35 | 5J8X | -6.12 | 0.23 |
| P0AES6 | 1AJ6 | NOV | -9.83 | 5L3J | -8.44 | 1.39 |
| P0AES6 | 5L3J | 6G9 | -6.96 | 1AJ6 | -7.11 | 0.15 |
| P0AEX9 | 4WMW | 3R6 | -5.36 | 4WMU | -5.34 | 0.02 |
| P0AEX9 | 4WMU | 19H | -9.59 | 4WMW | -7.08 | 2.51 |
| P0AGJ9 | 1VBM | YSA | -12.30 | 2YXN | -10.61 | 1.69 |
| P0AGJ9 | 2YXN | AZY | -8.22 | 1VBM | -7.79 | 0.43 |
| P0C6U8 | 4TWW | 3A7 | -7.34 | 4MDS | -7.43 | 0.09 |
| P0C6U8 | 4MDS | 23H | -7.70 | 4TWW | -7.26 | 0.44 |
| P0C6U8 | 2ALV | CY6 | -7.28 | 2ALV | -6.73 | 0.55 |
| P0C6U8 | 2ZU4 | ZU3 | -8.98 | 2ALV | -8.02 | 0.96 |
| P0C6U8 | 2ALV | CY6 | -7.28 | 3SZN | -6.84 | 0.44 |
| P0C6U8 | 3SZN | G75 | -6.81 | 2ZU4 | -7.50 | 0.69 |
| P0C6W3 | 4YOJ | RFM | -9.28 | 4YOI | -8.60 | 0.68 |
| P0C6W3 | 4YOI | 4F4 | -8.23 | 4YOJ | -8.67 | 0.44 |
| P0C6X7 | 2A5K | AZP | -7.26 | 4TWY | -7.28 | 0.02 |
| P0C6X7 | 4TWY | 3BL | -8.28 | 4TWY | -7.48 | 0.80 |
| P0C6X7 | 3D62 | 959 | -5.70 | 5N5O | -5.50 | 0.20 |
| P0C6X7 | 4TWY | 3BL | -8.28 | 3D62 | -7.29 | 0.99 |
| P0C6X7 | 5N5O | 8O5 | -8.42 | 3D62 | -6.63 | 1.79 |
| P0C6X7 | 3D62 | 959 | -5.70 | 2A5K | -5.37 | 0.33 |
| P0DMV8 | 5MKR | TI8 | -8.71 | 5BN9 | -8.73 | 0.02 |
| P0DMV8 | 5BN9 | ADP | -10.59 | 5MKR | -9.80 | 0.79 |
| P0DPI1 | 4HEV | AXM | -8.94 | 5V8U | -7.33 | 1.61 |
| P0DPI1 | 5V8U | 90M | -7.82 | 4HEV | -7.48 | 0.34 |
| P10153 | 2C02 | ADP | -7.87 | 1HI3 | -7.66 | 0.21 |
| P10153 | 1HI3 | A2P | -7.91 | 2C02 | -7.55 | 0.36 |
| P10408 | 2FSH | ANP | -8.26 | 2FSI | -6.70 | 1.56 |
| P10408 | 2FSI | ADP | -9.36 | 2FSH | -8.84 | 0.52 |
| P10584 | 2CVQ | NDP | -8.48 | 1BDM | -8.48 | 0.00 |
| P10584 | 1BDM | NAX | -9.39 | 2CVQ | -9.80 | 0.41 |
| P10613 | 5TZ1 | VT1 | -10.27 | 5V5Z | -10.47 | 0.20 |
| P10613 | 5V5Z | 1YN | -11.27 | 5TZ1 | -11.31 | 0.04 |
| P10632 | 2VN0 | TDZ | -10.94 | 2NNI | -10.85 | 0.09 |
| P10632 | 2NNI | MTK | -12.00 | 2VN0 | -10.98 | 1.02 |
| P10760 | 1XWF | ADN | -10.89 | 2H5L | -10.79 | 0.10 |
| P10760 | 2H5L | 3DD | -9.40 | 1XWF | -8.04 | 1.36 |
| P10824 | 5KDL | GSP | -14.09 | 1GDD | -12.74 | 1.35 |
| P10824 | 1GDD | GDP | -12.80 | 5KDL | -13.40 | 0.60 |
| P10828 | 2J4A | OEF | -11.20 | 1R6G | -6.18 | 5.02 |
| P10828 | 1R6G | 442 | -6.38 | 2J4A | -6.12 | 0.26 |
| P11021 | 5F2R | ACP | -11.49 | 3LDP | -11.06 | 0.43 |
| P11021 | 3LDP | 3P1 | -10.01 | 5F2R | -10.91 | 0.90 |
| P11086 | 2G72 | F21 | -10.21 | 3KR0 | -8.20 | 2.01 |
| P11086 | 3KR0 | 172 | -7.30 | 2G72 | -7.34 | 0.04 |
| P11142 | 6B1I | ADP | -10.88 | 3FZM | -9.87 | 1.01 |
| P11142 | 3FZM | 3GO | -11.53 | 6B1I | -10.98 | 0.55 |
| P11309 | 5TEX | 7AU | -10.11 | 4MBI | -10.31 | 0.20 |
| P11309 | 4MBI | 26K | -6.95 | 5TEX | -7.13 | 0.18 |
| P11712 | 5A5I | XI1 | -7.72 | 5K7K | -8.24 | 0.52 |
| P11712 | 5K7K | 6RJ | -8.68 | 5K7K | -8.33 | 0.35 |
| P11712 | 5K7K | 6RJ | -8.68 | 5A5J | -9.29 | 0.61 |
| P11712 | 5A5J | 6YF | -8.89 | 5A5I | -7.69 | 1.20 |
| P12499 | 5AH7 | C7J | -7.17 | 4K4P | -7.35 | 0.18 |
| P12499 | 4K4P | 3TL | -6.70 | 5AH7 | -6.13 | 0.57 |
| P12694 | 2BEU | THV | -8.54 | 1V1M | -7.28 | 1.26 |
| P12694 | 1V1M | TDP | -7.38 | 2BEU | -7.19 | 0.19 |
| P12821 | 4BZS | K26 | -10.03 | 6EN5 | -9.47 | 0.56 |
| P12821 | 6EN5 | BJ2 | -8.36 | 4BZS | -8.15 | 0.21 |
| P12823 | 1R6A | RVP | -6.96 | 2P1D | -7.06 | 0.1 |
| P12823 | 2P1D | 5GP | -7.74 | 1R6A | -7.61 | 0.13 |
| P13051 | 3FCI | 3FI | -7.90 | 2HXM | -7.53 | 0.37 |
| P13051 | 2HXM | 302 | -8.15 | 3FCI | -8.46 | 0.31 |
| P13551 | 4M1K | GDP | -8.41 | 2J7K | -8.86 | 0.45 |
| P13551 | 2J7K | GCP | -9.17 | 4M1K | -8.65 | 0.52 |
| P14210 | 5CT2 | CXS | -5.32 | 5CT3 | -5.45 | 0.13 |
| P14210 | 5CT3 | 54O | -4.14 | 5CT2 | -3.99 | 0.15 |
| P14555 | 3U8D | U8D | -9.18 | 1POE | -9.03 | 0.15 |
| P14555 | 1POE | GEL | -6.97 | 3U8D | -6.65 | 0.32 |
| P14618 | 4G1N | NZT | -8.63 | 3U2Z | -9.17 | 0.54 |
| P14618 | 3U2Z | 07T | -8.00 | 4G1N | -8.01 | 0.01 |
| P14677 | 1QMF | CES | -7.99 | 2ZC3 | -7.87 | 0.12 |
| P14677 | 2ZC3 | BMG | -6.90 | 2ZC3 | -7.41 | 0.51 |
| P14677 | 2ZC3 | BMG | -6.90 | 2Z2M | -7.49 | 0.59 |
| P14677 | 2Z2M | CDS | -8.95 | 1QMF | -8.25 | 0.7 |
| P14780 | 2OVZ | 5MR | -10.41 | 2OVX | -9.55 | 0.86 |
| P14780 | 2OVX | 4MR | -11.51 | 2OVZ | -10.62 | 0.89 |
| P14920 | 3ZNO | SE5 | -8.32 | 3ZNP | -8.22 | 0.10 |
| P14920 | 3ZNP | SE2 | -7.66 | 3ZNO | -7.44 | 0.22 |
| P15291 | 4EEM | UDP | -10.10 | 3EE5 | -9.68 | 0.42 |
| P15291 | 3EE5 | UDH | -9.50 | 4EEM | -9.57 | 0.07 |
| P15468 | 5ARK | UMP | -6.84 | 5ARL | -6.81 | 0.03 |
| P15468 | 5ARL | DCM | -7.00 | 5ARK | -7.64 | 0.64 |
| P15659 | 5IFD | 6AQ | -5.02 | 5IEQ | -5.72 | 0.70 |
| P15659 | 5IEQ | 1CI | -5.58 | 5IFD | -5.01 | 0.57 |
| P15790 | 4JR7 | GNP | -10.31 | 4FI1 | -10.45 | 0.14 |
| P15790 | 4FI1 | ATP | -9.99 | 4JR7 | -8.78 | 1.21 |
| P15917 | 1PWU | GM6 | -7.70 | 5D1T | -8.27 | 0.57 |
| P15917 | 5D1T | 56R | -9.43 | 1PWU | -7.92 | 1.51 |
| P16094 | 4YP2 | NCA | -6.49 | 1AHB | -5.79 | 0.70 |
| P16094 | 1AHB | FMP | -8.02 | 4YP2 | -6.93 | 1.09 |
| P16330 | 3ZBR | NAP | -10.53 | 2YDD | -9.99 | 0.54 |
| P16330 | 2YDD | 2AM | -7.82 | 3ZBR | -8.05 | 0.23 |
| P17252 | 3IW4 | LW4 | -11.23 | 4RA4 | -9.42 | 1.81 |
| P17252 | 4RA4 | 3KZ | -10.30 | 3IW4 | -10.48 | 0.18 |
| P17612 | 4UJA | 4L7 | -10.30 | 3OXT | -9.98 | 0.32 |
| P17612 | 3OXT | 3SB | -9.16 | 4UJA | -8.63 | 0.53 |
| P18031 | 1Q1M | 234 | -8.67 | 2FJN | -8.55 | 0.12 |
| P18031 | 1NZ7 | 901 | -6.78 | 1Q1M | -6.27 | 0.51 |
| P18031 | 2FJN | 73 | -10.95 | 3CWE | -10.05 | 0.90 |
| P18031 | 3EB1 | LZQ | -7.45 | 1NZ7 | -6.90 | 0.55 |
| P18031 | 3CWE | 825 | -10.35 | 2CNE | -8.28 | 2.07 |
| P18031 | 2CNE | DFJ | -10.11 | 3EB1 | 2.39 | 12.5 |
| P18946 | 3L74 | FMX | -12.21 | 3L71 | -10.42 | 1.79 |
| P18946 | 3L71 | AZO | -8.69 | 3L74 | -8.56 | 0.13 |
| P19483 | 1NBM | ATP | -9.14 | 1E79 | -9.15 | 0.01 |
| P19483 | 1W0K | ADP | -8.95 | 1W0K | -8.56 | 0.39 |
| P19483 | 1E79 | ADP | -8.63 | 2JIZ | -8.53 | 0.10 |
| P19483 | 2JIZ | ANP | -9.25 | 1NBM | -9.77 | 0.52 |
| P19491 | 3RTF | CWD | -8.78 | 5FHO | -8.29 | 0.49 |
| P19491 | 5FHO | 5XN | -10.66 | 3RTF | -7.30 | 3.36 |
| P19866 | 1RM3 | NDP | -9.44 | 1NBO | -9.59 | 0.15 |
| P19866 | 1NBO | NAD | -10.33 | 1RM3 | -8.93 | 1.40 |
| P20231 | 2BM2 | PM2 | -8.29 | 2FPZ | -8.07 | 0.22 |
| P20231 | 2FPZ | 270 | -6.48 | 2BM2 | -5.82 | 0.66 |
| P20339 | 3MJH | GTP | -12.89 | 1N6I | -12.99 | 0.10 |
| P20339 | 1N6I | GDP | -12.00 | 3MJH | -12.06 | 0.06 |
| P20701 | 3BQM | BQM | -10.49 | 4IXD | -8.30 | 2.19 |
| P20701 | 2ICA | 2IC | -10.20 | 2ICA | -8.62 | 1.58 |
| P20701 | 1XDG | AB8 | -8.10 | 1XDG | -8.09 | 0.01 |
| P20701 | 4IXD | 1HV | -9.82 | 3BQM | -7.15 | 2.67 |
| P20906 | 4JUC | TPP | -10.09 | 3FSJ | -8.94 | 1.15 |
| P20906 | 3FSJ | D7K | -9.89 | 4JUC | -10.56 | 0.67 |
| P21524 | 2EUD | GCQ | -9.14 | 2CVX | -8.46 | 0.68 |
| P21524 | 2CVX | ADP | -10.27 | 2EUD | -9.61 | 0.66 |
| P21673 | 2B58 | COA | -9.34 | 2FXF | -8.96 | 0.38 |
| P21673 | 2FXF | ACO | -10.07 | 2B58 | -8.52 | 1.55 |
| P21860 | 4OTW | DB8 | -7.93 | 3LMG | -8.36 | 0.43 |
| P21860 | 3LMG | ANP | -9.24 | 4OTW | -9.11 | 0.13 |
| P21873 | 3DVA | TPW | -8.34 | 3DUF | -7.85 | 0.49 |
| P21873 | 3DUF | R1T | -7.71 | 3DVA | -7.76 | 0.05 |
| P22102 | 4ZZ3 | 4DW | -8.95 | 4ZYT | -9.00 | 0.05 |
| P22102 | 4ZYT | 3Y9 | -9.46 | 4ZZ3 | -8.83 | 0.63 |
| P22102 | 1NJS | KEU | -11.15 | 1ZLY | -10.32 | 0.83 |
| P22102 | 1ZLY | DQB | -9.23 | 1NJS | -9.48 | 0.25 |
| P22629 | 1SRJ | NAB | -10.94 | 1N43 | -7.94 | 3.00 |
| P22629 | 1N43 | BTN | -7.19 | 1SRJ | -6.28 | 0.91 |
| P22734 | 3HVJ | 705 | -12.42 | 3NW9 | -8.66 | 3.76 |
| P22734 | 4PYL | TCW | -7.88 | 3HVJ | -7.59 | 0.29 |
| P22734 | 3NW9 | 637 | -13.64 | 5P8Y | -13.09 | 0.55 |
| P22734 | 5P8Y | 763 | -10.53 | 4PYL | -9.93 | 0.60 |
| P22894 | 5H8X | 5XT | -10.23 | 1ZP5 | -10.74 | 0.51 |
| P22894 | 1ZP5 | 2NI | -9.77 | 5H8X | -8.67 | 1.10 |
| P23458 | 6AAH | 9T6 | -10.52 | 5E1E | -9.82 | 0.70 |
| P23458 | 5E1E | 5JG | -8.90 | 6AAH | -8.89 | 0.01 |
| P23467 | 2H04 | 4UN | -9.38 | 2H03 | -8.76 | 0.62 |
| P23467 | 2H03 | 3UN | -7.71 | 2H04 | -7.43 | 0.28 |
| P23467 | 2I5X | UA5 | -8.01 | 2I4G | -7.43 | 0.58 |
| P23467 | 2I4G | UA1 | -7.94 | 2I5X | -8.64 | 0.70 |
| P23919 | 1E2G | TYD | -8.87 | 1NN3 | -9.38 | 0.51 |
| P23919 | 1NN3 | 2DT | -8.44 | 1E2G | -8.33 | 0.11 |
| P23946 | 1T31 | OHH | -11.45 | 2HVX | -9.51 | 1.94 |
| P23946 | 2HVX | DRX | -8.61 | 1T31 | -8.89 | 0.28 |
| P24941 | 3TNW | F18 | -7.61 | 2UZL | -7.74 | 0.13 |
| P24941 | 2UZL | C94 | -9.26 | 3TNW | -9.48 | 0.22 |
| P25440 | 4QEV | 31O | -8.26 | 4MR5 | -7.95 | 0.31 |
| P25440 | 4MR5 | 1K0 | -8.26 | 4QEV | -8.42 | 0.16 |
| P26281 | 6AN4 | J1F | -11.33 | 1EX8 | -10.68 | 0.65 |
| P26281 | 1EX8 | A4P | -11.21 | 6AN4 | -10.40 | 0.81 |
| P26662 | 4KTC | 1X3 | -12.04 | 4I33 | -11.74 | 0.30 |
| P26662 | 4I33 | 1BV | -12.39 | 4KTC | -11.66 | 0.73 |
| P26664 | 2GVF | NHN | -8.44 | 1RTL | -7.69 | 0.75 |
| P26664 | 1RTL | CPX | -7.80 | 2GVF | -6.47 | 1.33 |
| P27001 | 1B7Y | FYA | -11.83 | 2AKW | -7.96 | 3.87 |
| P27001 | 2AKW | 200 | -7.70 | 1B7Y | -6.30 | 1.40 |
| P27487 | 4N8E | 2KV | -8.85 | 4N8D | -8.76 | 0.09 |
| P27487 | 4N8D | 2KS | -8.55 | 4N8E | -9.32 | 0.77 |
| P27487 | 2G63 | AAF | -8.09 | 5T4H | -8.61 | 0.52 |
| P27487 | 5T4H | 75J | -9.64 | 2G63 | -8.39 | 1.25 |
| P27601 | 3CX8 | GSP | -13.53 | 1ZCB | -9.69 | 3.84 |
| P27601 | 1ZCB | GDP | -12.95 | 3CX8 | -12.90 | 0.05 |
| P27694 | 5E7N | 5KR | -6.69 | 4LUZ | -6.49 | 0.20 |
| P27694 | 4LUZ | 1XT | -8.79 | 5E7N | -8.06 | 0.73 |
| P27915 | 5E9Q | SAM | -9.00 | 3P8Z | -8.52 | 0.48 |
| P27915 | 3P8Z | 36A | -10.19 | 5E9Q | -9.79 | 0.40 |
| P27958 | 6BQK | Z1E | -9.74 | 2F9V | -7.53 | 2.21 |
| P27958 | 2F9V | BN6 | -7.26 | 6BQK | -7.50 | 0.24 |
| P28012 | 1JEP | DFL | -8.87 | 1FM8 | -9.66 | 0.79 |
| P28012 | 1FM8 | DDC | -9.52 | 1JEP | -9.36 | 0.16 |
| P28161 | 1HNC | GDN | -6.56 | 3GUR | -5.83 | 0.73 |
| P28161 | 3GUR | BYG | -6.74 | 1HNC | -6.66 | 0.08 |
| P28248 | 2V9X | DUT | -9.23 | 1XS4 | -8.49 | 0.74 |
| P28248 | 1XS4 | DCP | -8.40 | 2V9X | -7.27 | 1.13 |
| P28327 | 3C4Z | ADP | -8.98 | 4L9I | -9.11 | 0.13 |
| P28327 | 4L9I | 8PR | -8.58 | 3C4Z | -8.14 | 0.44 |
| P28523 | 3PWD | CZ0 | -9.79 | 1DAW | -10.49 | 0.70 |
| P28523 | 1DAW | ANP | -10.08 | 3PWD | -8.98 | 1.10 |
| P28845 | 4K1L | SFF | -8.61 | 4HFR | -7.83 | 0.78 |
| P28845 | 4HFR | 14M | -8.92 | 4K1L | -9.28 | 0.36 |
| P29317 | 5IA1 | ZZL | -9.89 | 5NKH | -9.20 | 0.69 |
| P29317 | 5NKH | 8ZQ | -10.52 | 5IA1 | -9.73 | 0.79 |
| P29375 | 5IVE | 6E8 | -9.04 | 5IVC | -6.67 | 2.37 |
| P29375 | 5IVC | 6E7 | -9.62 | 5IVE | -10.20 | 0.58 |
| P29474 | 5VVB | 9P7 | -9.73 | 5UOC | -10.12 | 0.39 |
| P29474 | 5UOC | 8FD | -10.56 | 5VVB | -10.62 | 0.06 |
| P29476 | 4IMW | 1EV | -10.82 | 3TYM | -11.17 | 0.35 |
| P29476 | 3TYM | 08R | -9.09 | 4IMW | -8.75 | 0.34 |
| P29477 | 1QW4 | 3AR | -6.08 | 3EBF | -5.20 | 0.88 |
| P29477 | 4UX6 | YWO | -10.33 | 1QW4 | -10.39 | 0.06 |
| P29477 | 2Y37 | A54 | -8.36 | 2Y37 | -8.87 | 0.51 |
| P29477 | 3EBF | 332 | -9.93 | 4UX6 | -9.70 | 0.23 |
| P29597 | 4GJ3 | 0XP | -8.70 | 4GIH | -8.02 | 0.68 |
| P29597 | 6AAM | 9T6 | -9.43 | 4GJ3 | -9.80 | 0.37 |
| P29597 | 6DBM | G4J | -10.29 | 4WOV | -8.82 | 1.47 |
| P29597 | 4GIH | 0X5 | -8.05 | 6AAM | -7.76 | 0.29 |
| P29597 | 5C03 | AGS | -9.55 | 5C03 | -9.87 | 0.32 |
| P29597 | 4WOV | 3SM | -8.94 | 6DBM | -8.73 | 0.21 |
| P30405 | 5CCR | 4ZT | -6.92 | 5CBW | -6.61 | 0.31 |
| P30405 | 5CBW | 4ZO | -6.89 | 5CCR | -6.85 | 0.04 |
| P30419 | 4C2Z | 646 | -10.49 | 3IU2 | -8.78 | 1.71 |
| P30419 | 3IU2 | 96 | -9.09 | 4C2Z | -8.88 | 0.21 |
| P31013 | 6ECG | PM9 | -8.95 | 2YCN | -8.28 | 0.67 |
| P31013 | 2YCN | P61 | -9.93 | 6ECG | -9.75 | 0.18 |
| P31749 | 3MVH | WFE | -10.14 | 4EKK | -9.38 | 0.76 |
| P31749 | 3QKM | SM9 | -10.72 | 4EKK | -9.43 | 1.29 |
| P31749 | 4EKK | ANP | -9.28 | 3CQW | -8.93 | 0.35 |
| P31749 | 3CQW | CQW | -8.94 | 3QKM | -8.28 | 0.66 |
| P31749 | 4EKK | ANP | -8.78 | 3QKM | -9.06 | 0.28 |
| P31749 | 3QKM | SM9 | -10.72 | 3MVH | -9.85 | 0.87 |
| P31751 | 2UW9 | GVP | -9.87 | 3E87 | -8.88 | 0.99 |
| P31751 | 3E87 | G95 | -8.90 | 2UW9 | -9.42 | 0.52 |
| P33590 | 3MZ9 | BHN | -7.64 | 5ON4 | -7.15 | 0.49 |
| P33590 | 5ON4 | 9YK | -6.88 | 3MZ9 | -7.45 | 0.57 |
| P33981 | 4O6L | 2QK | -10.18 | 4JT3 | -9.90 | 0.28 |
| P33981 | 5NTT | SVE | -10.15 | 4O6L | -9.61 | 0.54 |
| P33981 | 5N9S | 8QW | -12.28 | 5N9S | -9.28 | 3.00 |
| P33981 | 4JT3 | 1PH | -10.06 | 5NTT | -9.08 | 0.98 |
| P34913 | 3I28 | 34N | -11.59 | 3KOO | -10.82 | 0.77 |
| P34913 | 3KOO | 24D | -8.96 | 3I28 | -9.71 | 0.75 |
| P35557 | 3ID8 | MRK | -7.80 | 4IWV | -8.29 | 0.49 |
| P35557 | 4IWV | 1J9 | -10.77 | 3ID8 | -8.14 | 2.63 |
| P35747 | 5DQF | CZE | -7.19 | 5ID9 | -7.87 | 0.68 |
| P35747 | 5ID9 | 6A4 | -6.84 | 5DQF | -6.32 | 0.52 |
| P35968 | 2QU6 | 857 | -10.80 | 3VNT | -10.84 | 0.04 |
| P35968 | 3VNT | 0JA | -12.59 | 2QU6 | -8.75 | 3.84 |
| P36897 | 5USQ | 8LY | -10.85 | 3TZM | -10.25 | 0.60 |
| P36897 | 3TZM | 85 | -11.27 | 5USQ | -10.21 | 1.06 |
| P37231 | 4HEE | 14R | -12.58 | 3TY0 | -10.74 | 1.84 |
| P37231 | 2VV1 | 4HD | -9.97 | 4HEE | -8.08 | 1.89 |
| P37231 | 4R6S | 3K2 | -12.34 | 4HEE | -11.00 | 1.34 |
| P37231 | 4HEE | 14R | -12.58 | 4R6S | -9.72 | 2.86 |
| P37231 | 6-Aug | BXG | -8.96 | 4R6S | -8.68 | 0.28 |
| P37231 | 5Z6S | RTF | -10.01 | 2VV1 | -9.83 | 0.18 |
| P37231 | 6AVI | GW9 | -7.97 | 5DV3 | -7.73 | 0.24 |
| P37231 | 3TY0 | 82 | -11.17 | 6-Aug | -10.48 | 0.69 |
| P37231 | 5DV3 | B05 | -9.42 | 6AVI | -8.56 | 0.86 |
| P37231 | 4R6S | 3K2 | -11.41 | 5Z6S | -9.75 | 1.66 |
| P37268 | 3WCI | BH5 | -7.57 | 3WCH | -7.57 | 0.00 |
| P37268 | 3WCH | 8PH | -7.39 | 3WCI | -6.94 | 0.45 |
| P37344 | 2C99 | ANP | -9.79 | 4QOS | -9.48 | 0.31 |
| P37344 | 4QOS | ADP | -8.86 | 2C99 | -8.98 | 0.12 |
| P39058 | 2VAV | CSC | -8.59 | 2VAT | -7.35 | 1.24 |
| P39058 | 2VAT | COA | -8.43 | 2VAV | -8.43 | 0.00 |
| P40233 | 2CSN | CKI | -6.80 | 1CSN | -7.63 | 0.83 |
| P40233 | 1CSN | ATP | -9.62 | 2CSN | -8.41 | 1.21 |
| P41222 | 3O2Y | PLM | -6.30 | 4IMN | -6.16 | 0.14 |
| P41222 | 4IMN | 1PG | -4.40 | 3O2Y | -4.41 | 0.01 |
| P41279 | 4Y83 | 49B | -10.52 | 4Y85 | -9.27 | 1.25 |
| P41279 | 4Y85 | 499 | -11.18 | 4Y83 | -9.23 | 1.95 |
| P41743 | 5LI1 | ANP | -10.70 | 5LI9 | -7.90 | 2.8 |
| P41743 | 5LI9 | ACP | -10.68 | 5LI1 | -10.57 | 0.11 |
| P42216 | 1GQC | CMK | -10.17 | 1GQC | -9.53 | 0.64 |
| P42216 | 1GQC | C5P | -8.19 | 1GQC | -8.25 | 0.06 |
| P42330 | 4FAL | 0T0 | -10.89 | 4FA3 | -10.17 | 0.72 |
| P42330 | 6F78 | CVN | -10.10 | 4FAL | -10.02 | 0.08 |
| P42330 | 4FA3 | 0SL | -10.56 | 5JM5 | -9.96 | 0.6 |
| P42330 | 4WDW | WDW | -8.56 | 6F78 | -8.50 | 0.06 |
| P42330 | 5JM5 | 6LG | -9.51 | 1RY8 | -9.92 | 0.41 |
| P42330 | 1RY8 | RUT | -12.86 | 4WDW | -12.67 | 0.19 |
| P42336 | 5UL1 | 8DY | -8.29 | 4WAF | -8.44 | 0.15 |
| P42336 | 4WAF | 3K6 | -9.84 | 5UL1 | -8.94 | 0.90 |
| P42574 | 1NMS | 161 | -7.59 | 1NMQ | -6.09 | 1.50 |
| P42574 | 1NMQ | 160 | -6.81 | 1NMS | -6.77 | 0.04 |
| P43405 | 4YJV | 4DT | -9.30 | 3FQH | -9.77 | 0.47 |
| P43405 | 5Y5U | 8OU | -9.63 | 4YJV | -9.07 | 0.56 |
| P43405 | 3FQH | 057 | -8.82 | 5CXH | -8.72 | 0.10 |
| P43405 | 5CXH | 55M | -8.80 | 5Y5U | -7.95 | 0.85 |
| P43889 | 2V0J | H2U | -8.53 | 4E1K | -7.29 | 1.24 |
| P43889 | 4E1K | 0N5 | -10.22 | 2V0J | -8.69 | 1.53 |
| P45452 | 3ZXH | E41 | -7.90 | 2YIG | -7.97 | 0.07 |
| P45452 | 2YIG | 5EL | -11.38 | 3ZXH | -10.22 | 1.16 |
| P47811 | 2GHL | LIB | -9.58 | 4TYH | -9.68 | 0.10 |
| P47811 | 5O90 | SB4 | -9.81 | 2GHL | -8.18 | 1.63 |
| P47811 | 2EWA | SB2 | -10.89 | 2EWA | -8.21 | 2.68 |
| P47811 | 4TYH | 39G | -8.93 | 5O90 | -8.72 | 0.21 |
| P47934 | 1NDI | COA | -8.09 | 2H3P | -8.24 | 0.15 |
| P47934 | 2H3P | ACO | -8.46 | 1NDI | -7.82 | 0.64 |
| P48730 | 5OKT | 9XK | -8.66 | 4TWC | -8.43 | 0.23 |
| P48730 | 5IH6 | AUG | -8.74 | 5OKT | -9.36 | 0.62 |
| P48730 | 4TWC | 37J | -9.68 | 5IH5 | -9.20 | 0.48 |
| P48730 | 5IH5 | AUE | -9.69 | 5IH6 | -8.95 | 0.74 |
| P48735 | 6ADI | NDP | -10.39 | 5SVO | -8.63 | 1.76 |
| P48735 | 5SVO | NAP | -9.44 | 6ADI | -9.50 | 0.06 |
| P49336 | 4F6W | 0SS | -11.01 | 4F6S | -10.18 | 0.83 |
| P49336 | 4F6S | 0SQ | -9.50 | 4F6W | -8.33 | 1.17 |
| P49336 | 4F7J | 0SU | -10.11 | 4F70 | -8.54 | 1.57 |
| P49336 | 4F70 | 0ST | -9.62 | 4F7J | -9.53 | 0.09 |
| P49610 | 4AZG | OAN | -7.33 | 4AZH | -7.90 | 0.57 |
| P49610 | 4AZH | LOG | -6.54 | 4AZG | -6.21 | 0.33 |
| P49841 | 3ZDI | UGJ | -8.34 | 4DIT | -7.35 | 0.99 |
| P49841 | 3F88 | 3HT | -9.31 | 3F88 | -8.39 | 0.92 |
| P49841 | 4DIT | 0KD | -8.65 | 4NM0 | -7.63 | 1.02 |
| P49841 | 4NM0 | ADP | -8.34 | 3ZDI | -8.33 | 0.01 |
| P49902 | 2XJE | ATP | -7.72 | 4H4B | -7.46 | 0.26 |
| P49902 | 2XJC | 5GP | -9.56 | 2XJC | -9.56 | 0.00 |
| P49902 | 2XJE | ATP | -9.72 | 2XJE | -9.26 | 0.46 |
| P49902 | 6DDH | IMP | -9.02 | 2XJE | -9.46 | 0.44 |
| P49902 | 5OPM | DTP | -7.72 | 5OPM | -7.43 | 0.29 |
| P49902 | 4H4B | 11H | -8.99 | 6DDH | -8.40 | 0.59 |
| P49789 | 3FIT | A | -7.82 | 6FIT | -6.24 | 1.58 |
| P48789 | 6FIT | AMW | -8.23 | 3FIT | -6.77 | 1.46 |
| P50225 | 2D06 | EST | -10.71 | 3U3K | -8.36 | 2.35 |
| P50225 | 3U3K | 03V | -7.76 | 2D06 | -7.65 | 0.11 |
| P50579 | 2ADU | R20 | -7.24 | 1YW7 | -7.13 | 0.11 |
| P50579 | 1YW7 | A41 | -8.26 | 2ADU | -7.62 | 0.64 |
| P50750 | 3LQ5 | SLQ | -8.14 | 3TN8 | -8.95 | 0.81 |
| P50750 | 3TN8 | F18 | -8.03 | 3LQ5 | -6.91 | 1.12 |
| P51149 | 1T91 | GTP | -14.22 | 3LAW | -13.10 | 1.12 |
| P51149 | 3LAW | GNP | -13.79 | 1T91 | -13.69 | 0.10 |
| P51449 | 4WPF | 3SN | -10.95 | 4QM0 | -9.48 | 1.47 |
| P51449 | 6BN6 | XGH | -11.40 | 4WPF | -10.84 | 0.56 |
| P51449 | 6CN6 | F7J | -14.23 | 6CN6 | -10.39 | 3.84 |
| P51449 | 4QM0 | 39K | -10.29 | 6BN6 | -8.88 | 1.41 |
| P51857 | 3CAS | ASD | -11.68 | 3UZX | -10.80 | 0.88 |
| P51857 | 3UZX | AOM | -12.29 | 3CAS | -11.71 | 0.58 |
| P51955 | 2XKF | BX1 | -8.67 | 2W5B | -7.62 | 1.05 |
| P51955 | 2W5B | AGS | -8.24 | 2XKF | -8.00 | 0.24 |
| P52333 | 4QPS | 37Q | -9.77 | 4HVG | -9.00 | 0.77 |
| P52333 | 4HVG | 19Q | -7.53 | 4QPS | -8.06 | 0.53 |
| P52699 | 5EWA | 9BZ | -5.64 | 5HH4 | -5.56 | 0.08 |
| P52699 | 5HH4 | 60M | -6.97 | 5EWA | -5.84 | 1.13 |
| P52700 | 2GFJ | VI | -7.68 | 2FU9 | -7.27 | 0.41 |
| P52700 | 2FU9 | MP2 | -6.52 | 2GFJ | -6.47 | 0.05 |
| P52732 | 5ZO8 | 4C5 | -10.54 | 2GM1 | -10.36 | 0.18 |
| P52732 | 2GM1 | 2AZ | -11.27 | 5ZO8 | -8.90 | 2.37 |
| P53355 | 2W4K | ADP | -8.36 | 5AUT | -8.19 | 0.17 |
| P53355 | 5AUT | 2AN | -8.91 | 2W4K | -8.30 | 0.61 |
| P53582 | 5YKP | OVA | -7.14 | 4HXX | -6.45 | 0.69 |
| P53582 | 4HXX | 1AY | -9.01 | 5YKP | -9.33 | 0.32 |
| P53779 | 1PMN | 984 | -9.98 | 4U79 | -8.41 | 1.57 |
| P53779 | 3FV8 | JK3 | -8.26 | 1PMN | -6.88 | 1.38 |
| P53779 | 4U79 | 3EL | -11.24 | 6EKD | -11.70 | 0.46 |
| P53779 | 6EKD | B9K | -9.67 | 3FV8 | -8.56 | 1.11 |
| P55055 | 1PQ6 | 965 | -13.02 | 4DK8 | -10.09 | 2.93 |
| P55055 | 4DK8 | 0KT | -10.34 | 1PQ6 | -6.15 | 4.19 |
| P56221 | 3STD | MQ0 | -12.29 | 4STD | -9.46 | 2.83 |
| P56221 | 4STD | BFS | -10.42 | 3STD | -9.53 | 0.89 |
| P56273 | 4J7D | I31 | -8.87 | 4LWV | -8.12 | 0.75 |
| P56273 | 4LWV | 20W | -9.38 | 4J7D | -8.36 | 1.02 |
| P60045 | 1TD7 | NFL | -7.73 | 3NJU | -7.48 | 0.25 |
| P60045 | 3NJU | ANN | -5.97 | 1TD7 | -6.16 | 0.19 |
| P60568 | 1PY2 | FRH | -9.28 | 1PW6 | -7.51 | 1.77 |
| P60568 | 1PW6 | FRB | -8.80 | 1PY2 | -8.95 | 0.15 |
| P60766 | 3EG5 | GNP | -12.60 | 5C2J | -11.05 | 1.55 |
| P60766 | 5C2J | GDP | -9.87 | 3EG5 | -10.13 | 0.26 |
| P61157 | 2P9U | ANP | -10.30 | 2P9P | -10.43 | 0.13 |
| P61157 | 2P9P | ADP | -10.35 | 2P9U | -10.40 | 0.05 |
| P61586 | 4XOI | GTP | -12.30 | 1S1C | -12.30 | 0.00 |
| P61586 | 1S1C | GNP | -12.86 | 4XOI | -12.40 | 0.46 |
| P62483 | 3EB4 | NDP | -15.16 | 2R9R | -9.49 | 5.67 |
| P62483 | 2R9R | NAP | -14.90 | 3EB4 | -15.14 | 0.24 |
| P62491 | 5C46 | GSP | -13.41 | 1OIX | -13.29 | 0.12 |
| P62491 | 1OIX | GDP | -12.82 | 5C46 | -12.64 | 0.18 |
| P62508 | 2ZAS | 1OH | -9.89 | 2GPP | -8.63 | 1.26 |
| P62508 | 2GPP | 1BA | -11.36 | 2ZAS | -7.30 | 4.06 |
| P62575 | 2YA7 | ZMR | -8.95 | 5KKY | -8.01 | 0.94 |
| P62575 | 5KKY | 6UD | -8.71 | 2YA7 | -8.78 | 0.07 |
| P62593 | 1AXB | FOS | -6.95 | 1JWZ | -6.83 | 0.12 |
| P62593 | 1JWZ | 105 | -7.90 | 1AXB | -8.18 | 0.28 |
| P62617 | 1H48 | C5P | -6.40 | 2AMT | -5.67 | 0.73 |
| P62617 | 2AMT | 1AA | -5.66 | 1H48 | -5.89 | 0.23 |
| P62937 | 5T9U | 7HG | -8.76 | 5T9W | -8.80 | 0.04 |
| P62937 | 5T9W | 78E | -9.20 | 5T9U | -8.96 | 0.24 |
| P62993 | 3IN7 | AYQ | -8.53 | 3IMJ | -7.61 | 0.92 |
| P62993 | 3IMJ | AYI | -6.85 | 3IN7 | -7.72 | 0.87 |
| P63000 | 3SBE | GNP | -9.64 | 1I4D | -10.33 | 0.69 |
| P63000 | 1I4D | GDP | -11.61 | 3SBE | -11.56 | 0.05 |
| P63086 | 4N4S | 2H1 | -12.78 | 4N4S | -8.32 | 4.46 |
| P63086 | 4XOY | DX4 | -4.87 | 4N4S | -5.19 | 0.32 |
| P63086 | 4GVA | ADP | -7.61 | 4GVA | -8.47 | 0.86 |
| P63086 | 4N4S | 2H1 | -12.41 | 4XOY | -9.92 | 2.49 |
| P63820 | 4NAU | 2W3 | -9.24 | 4NAH | -9.31 | 0.07 |
| P63820 | 4NAH | 2VJ | -8.92 | 4NAU | -8.20 | 0.72 |
| P65248 | 2CCG | TMP | -8.69 | 4GFD | -7.17 | 1.52 |
| P65248 | 4GFD | 0YB | -9.43 | 2CCG | -8.96 | 0.47 |
| P67091 | 4R2L | ATP | -10.75 | 4R2M | -10.04 | 0.71 |
| P67091 | 4R2M | ANP | -10.06 | 4R2L | -10.04 | 0.02 |
| P69834 | 5MRP | 6BC | -11.08 | 4NAN | -7.66 | 3.42 |
| P69834 | 4NAN | 2JM | -7.68 | 5MRP | -5.64 | 2.04 |
| P76143 | 3GND | 5RP | -6.60 | 4P2V | -6.66 | 0.06 |
| P76143 | 4P2V | 26T | -6.41 | 3GND | -6.28 | 0.13 |
| P78536 | 3LEA | Z93 | -9.30 | 3LGP | -8.98 | 0.32 |
| P78536 | 3LGP | 50X | -12.74 | 3LEA | -9.06 | 3.68 |
| P80561 | 1TF9 | PHI | -7.22 | 1XBU | -6.79 | 0.43 |
| P80561 | 1XBU | IOY | -6.63 | 1TF9 | -7.11 | 0.48 |
| P82597 | 4KE8 | 1QY | -5.92 | 4KE7 | -5.50 | 0.42 |
| P82597 | 4KE7 | 1QX | -5.65 | 4KE8 | -5.99 | 0.34 |
| P93330 | 4GY9 | ZIP | -6.01 | 4JHI | -6.18 | 0.17 |
| P93330 | 4JHI | EMU | -7.15 | 4GY9 | -7.08 | 0.07 |
| P93836 | 1TFZ | 869 | -5.60 | 1TG5 | -6.46 | 0.86 |
| P93836 | 1TG5 | 645 | -6.73 | 1TFZ | -6.11 | 0.62 |
| P95607 | 3HGI | BEZ | -6.05 | 3I4Y | -5.74 | 0.31 |
| P95607 | 3I4Y | 35C | -6.48 | 3HGI | -5.57 | 0.91 |
| P96907 | 4FNB | 3HC | -5.95 | 4FND | -5.14 | 0.81 |
| P96907 | 4FND | 3H9 | -6.98 | 4FNB | -6.12 | 0.86 |
| P97612 | 3K84 | K84 | -8.86 | 3K83 | -8.42 | 0.44 |
| P97612 | 3K83 | F27 | -11.61 | 3K84 | -10.13 | 1.48 |
| P98170 | 6EY2 | C3T | -8.23 | 5C3K | -7.05 | 1.18 |
| P98170 | 5C3K | 4XF | -6.24 | 6EY2 | -6.42 | 0.18 |
| P9WFX1 | 3RV6 | VAE | -9.60 | 3RV9 | -8.39 | 1.21 |
| P9WFX1 | 3RV9 | RVD | -6.96 | 3RV6 | -7.64 | 0.68 |
| P9WGR0 | 5VRN | 9JM | -11.12 | 5VRM | -12.05 | 0.93 |
| P9WGR0 | 5VRM | 9JJ | -12.42 | 5VRN | -10.82 | 1.60 |
| P9WGR1 | 4D0S | 9G4 | -8.98 | 4OXY | -9.61 | 0.63 |
| P9WGR1 | 4OXY | 1TN | -7.80 | 4D0S | -7.88 | 0.08 |
| P9WHE9 | 1W19 | T1P | -8.14 | 2C9D | -7.81 | 0.33 |
| P9WHE9 | 2C9D | PHR | -7.15 | 1W19 | -7.58 | 0.43 |
| P9WI81 | 3F69 | XDR | -14.37 | 5U94 | -11.41 | 2.96 |
| P9WI81 | 5U94 | G93 | -10.03 | 3F69 | -8.95 | 1.08 |
| P9WIL5 | 2A84 | ATP | -11.38 | 3ISJ | -10.35 | 1.03 |
| P9WIL5 | 3ISJ | A8D | -8.16 | 2A84 | -7.14 | 1.02 |
| P9WMC0 | 5F0C | 5TE | -11.77 | 5F0H | -11.03 | 0.74 |
| P9WMC0 | 5F0H | 5TC | -13.31 | 5F0C | -10.40 | 2.91 |
| P9WMC1 | 5F04 | 5TB | -9.64 | 4M3D | -9.65 | 0.01 |
| P9WMC1 | 4M3D | 2H2 | -12.22 | 5F04 | -11.35 | 0.87 |
| P9WMC1 | 5J3L | 6FR | -8.57 | 5F04 | -7.30 | 1.27 |
| P9WMC1 | 5F04 | 5TB | -9.64 | 5J3L | -10.47 | 0.83 |
| P9WNH5 | 2WUG | HPK | -7.59 | 5JZB | -7.07 | 0.52 |
| P9WNH5 | 5JZB | 6OT | -7.29 | 2WUG | -5.63 | 1.66 |
| P9WNX1 | 4KM2 | ATR | -9.86 | 2CIG | -9.06 | 0.80 |
| P9WNX1 | 2CIG | 1DG | -13.08 | 4KM2 | -11.34 | 1.74 |
| P9WNX1 | 5U26 | MMV | -7.86 | 6DDP | -8.26 | 0.4 |
| P9WNX1 | 6DDP | G6Y | -10.34 | 5U26 | -10.66 | 0.32 |
| P9WQ81 | 4WYD | 3VR | -6.22 | 4MQQ | -6.13 | 0.09 |
| P9WQ81 | 4MQQ | 2B6 | -9.05 | 4WYD | -8.67 | 0.38 |
| Q00441 | 1JIN | KTN | -8.16 | 1OXA | -8.22 | 0.06 |
| Q00441 | 1OXA | DEB | -8.73 | 1JIN | -9.04 | 0.31 |
| Q00610 | 4G55 | VH2 | -8.62 | 2XZG | -8.10 | 0.52 |
| Q00610 | 2XZG | VH1 | -9.60 | 4G55 | -9.25 | 0.35 |
| Q00955 | 5CTB | 57J | -8.88 | 3TVW | -9.28 | 0.40 |
| Q00955 | 3TVW | 07H | -8.58 | 5CTB | -7.85 | 0.73 |
| Q00972 | 4E01 | ANP | -10.13 | 3TZ5 | -11.07 | 0.94 |
| Q00972 | 3TZ5 | ADP | -10.59 | 4E01 | -9.93 | 0.66 |
| Q00987 | 5LAY | 6SS | -9.10 | 4ZYF | -9.87 | 0.77 |
| Q00987 | 4ZYF | 4T4 | -10.21 | 5LAY | -9.57 | 0.64 |
| Q01064 | 5B25 | 4QJ | -12.50 | 5W6E | -9.91 | 2.59 |
| Q01064 | 5W6E | 0NY | -8.61 | 5B25 | -7.61 | 1.00 |
| Q02127 | 2B0M | 201 | -11.22 | 5HIN | -10.67 | 0.55 |
| Q02127 | 5HIN | 1KL | -10.66 | 2B0M | -8.74 | 1.92 |
| Q02293 | 4GTO | 7TO | -8.10 | 1O1S | -8.88 | 0.78 |
| Q02293 | 1O1S | 1NH | -8.15 | 4GTO | -7.82 | 0.33 |
| Q03181 | 3SP9 | IL2 | -10.06 | 5U44 | -8.87 | 1.19 |
| Q03181 | 5U44 | 7SV | -10.56 | 3SP9 | -10.36 | 0.20 |
| Q05097 | 5MIH | 7NU | -6.15 | 4YW7 | -4.95 | 1.20 |
| Q05097 | 4YW7 | 4J0 | -7.01 | 5MIH | -6.95 | 0.06 |
| Q05769 | 1DDX | PGX | -9.48 | 3MDL | -8.80 | 0.68 |
| Q05769 | 3OLU | 1AG | -8.50 | 3OLU | -9.11 | 0.61 |
| Q05769 | 3MDL | 1AG | -7.67 | 5FDQ | -7.53 | 0.14 |
| Q05769 | 5FDQ | 60A | -4.50 | 1DDX | -4.43 | 0.07 |
| Q06187 | 4ZLZ | 4RV | -8.84 | 4NWM | -8.11 | 0.73 |
| Q06187 | 4NWM | 2P5 | -11.50 | 4ZLZ | -11.19 | 0.31 |
| Q06528 | 5JR3 | 4MU | -7.28 | 4WXH | -7.20 | 0.08 |
| Q06528 | 4WXH | 3VL | -13.10 | 5JR3 | -10.45 | 2.65 |
| Q07343 | 2QYL | NPV | -10.55 | 5K6J | -10.25 | 0.30 |
| Q07343 | 5K6J | 6QQ | -8.43 | 2QYL | -8.19 | 0.24 |
| Q07817 | 3ZLO | X8U | -12.57 | 3ZLN | -10.85 | 1.72 |
| Q07817 | 3ZLN | H0Y | -12.17 | 3ZLO | -11.75 | 0.42 |
| Q07820 | 4OQ5 | 2UU | -12.01 | 4HW2 | -11.95 | 0.06 |
| Q07820 | 4HW2 | 19H | -10.26 | 4OQ5 | -7.29 | 2.97 |
| Q07869 | 1K7L | 544 | -10.66 | 3VI8 | -10.57 | 0.09 |
| Q07869 | 3VI8 | 13M | -11.45 | 1K7L | -10.47 | 0.98 |
| Q08210 | 4CQA | ID6 | -11.25 | 1TV5 | -8.90 | 2.35 |
| Q08210 | 1TV5 | A26 | -8.90 | 4CQA | -8.26 | 0.64 |
| Q08499 | 5K1I | 6PT | -8.77 | 4W1O | -8.33 | 0.44 |
| Q08499 | 4W1O | 3GJ | -8.49 | 5K1I | -8.45 | 0.04 |
| Q08499 | 3SL8 | JN7 | -8.77 | 3IAK | -7.59 | 1.18 |
| Q08499 | 3IAK | EV1 | -7.91 | 3SL8 | -7.88 | 0.03 |
| Q08603 | 3C72 | CX1 | -9.38 | 3PZ1 | -9.00 | 0.38 |
| Q08603 | 3PZ1 | 3PZ | -8.03 | 3C72 | -8.58 | 0.55 |
| Q12051 | 2ZEU | B71 | -8.52 | 2Z4W | -7.27 | 1.25 |
| Q12051 | 2Z4W | 749 | -8.72 | 2ZEU | -8.99 | 0.27 |
| Q12341 | 4PSX | COA | -8.45 | 1BOB | -7.78 | 0.67 |
| Q12341 | 1BOB | ACO | -7.44 | 4PSX | -8.24 | 0.8 |
| Q12852 | 5CEP | 50E | -8.60 | 5CEO | -8.76 | 0.16 |
| Q12852 | 5CEO | 50D | -9.20 | 5CEP | -8.23 | 0.97 |
| Q13231 | 1HKM | ALI | -6.30 | 5NRA | -6.56 | 0.26 |
| Q13231 | 5NRA | 95K | -8.77 | 1HKM | -8.92 | 0.15 |
| Q13546 | 6C4D | EJP | -13.60 | 5HX6 | -11.84 | 1.76 |
| Q13546 | 5HX6 | 65U | -12.14 | 6C4D | -11.93 | 0.21 |
| Q13627 | 4MQ2 | 2C4 | -9.45 | 4MQ1 | -9.41 | 0.04 |
| Q13627 | 4MQ1 | 2C3 | -10.76 | 4MQ2 | -10.14 | 0.62 |
| Q13882 | 5DA3 | 58V | -9.85 | 5H2U | -10.14 | 0.29 |
| Q13882 | 5H2U | 1N1 | -10.90 | 5DA3 | -8.07 | 2.83 |
| Q13976 | 4QX5 | CMP | -9.99 | 5J48 | -9.55 | 0.44 |
| Q13976 | 5J48 | 6FW | -11.56 | 4QX5 | -9.54 | 2.02 |
| Q14397 | 4PX3 | 2WX | -11.89 | 4OHK | -11.26 | 0.63 |
| Q14397 | 4OHK | 2TE | -11.47 | 4PX3 | -10.93 | 0.54 |
| Q14416 | 5CNJ | 52Q | -9.23 | 4XAS | -9.19 | 0.04 |
| Q14416 | 4XAS | 40H | -9.83 | 5CNJ | -9.12 | 0.71 |
| Q15119 | 2BU8 | ADP | -8.62 | 5M4N | -6.92 | 1.70 |
| Q15119 | 5M4N | 7FV | -10.18 | 2BU8 | -10.83 | 0.65 |
| Q16620 | 4AT4 | T6E | -12.86 | 4AT5 | -10.99 | 1.87 |
| Q16620 | 4AT5 | MUJ | -10.70 | 4AT4 | -9.04 | 1.66 |
| Q16651 | 3E16 | B4C | -8.21 | 3FVF | -8.56 | 0.35 |
| Q16651 | 3FVF | 1JZ | -6.40 | 3E16 | -6.50 | 0.10 |
| Q16769 | 2AFZ | NVI | -3.81 | 2AFX | -4.07 | 0.26 |
| Q16769 | 2AFX | 1BN | -6.50 | 2AFZ | -6.29 | 0.21 |
| Q1R2J4 | 5AAP | VNY | -9.02 | 5AAL | -8.24 | 0.78 |
| Q1R2J4 | 5AAL | 8L8 | -9.45 | 5AAP | -9.11 | 0.34 |
| Q21A49 | 5JFM | COA | -7.07 | 5JFM | -6.93 | 0.14 |
| Q21A49 | 5JFM | 1VU | -7.33 | 5JFM | -7.02 | 0.31 |
| Q27895 | 4KH6 | AU1 | -10.33 | 4KH4 | -10.53 | 0.20 |
| Q27895 | 4KH4 | ANP | -11.63 | 4KH6 | -10.83 | 0.80 |
| Q2LG68 | 4CB7 | 41G | -8.55 | 4CB6 | -8.80 | 0.25 |
| Q2LG68 | 4CB6 | 29R | -7.03 | 4CB7 | -6.62 | 0.41 |
| Q2MG72 | 5U1E | 9CS | -9.46 | 5U1I | -9.59 | 0.13 |
| Q2MG72 | 5U1I | 7QM | -9.73 | 5U1E | -9.53 | 0.20 |
| Q2RSB2 | 2OO5 | TXD | -11.63 | 1U2G | -11.43 | 0.20 |
| Q2RSB2 | 1U2G | APR | -10.60 | 2OO5 | -9.50 | 1.10 |
| Q2YY41 | 3F0X | 53T | -10.64 | 3F0Q | -10.81 | 0.17 |
| Q2YY41 | 3F0Q | 52V | -10.36 | 3F0X | -10.23 | 0.13 |
| Q385E8 | 2X2N | X2N | -10.21 | 4G3J | -10.67 | 0.46 |
| Q385E8 | 4G3J | VNT | -10.17 | 2X2N | -9.86 | 0.31 |
| Q389T8 | 2WP6 | WP6 | -9.66 | 6BU7 | -6.02 | 3.64 |
| Q389T8 | 6BU7 | RD0 | -8.15 | 2WP6 | -8.17 | 0.02 |
| Q3F0V8 | 4KGK | GTP | -9.52 | 4KGM | -9.59 | 0.07 |
| Q3F0V8 | 4KGM | ATP | -9.67 | 4KGK | -9.92 | 0.25 |
| Q3JP94 | 3GQT | UFO | -6.61 | 3EON | -6.36 | 0.25 |
| Q3JP94 | 3EON | 341 | -4.75 | 3GQT | -4.73 | 0.02 |
| Q3JRA0 | 3MBM | CYT | -4.37 | 3F0G | -4.18 | 0.19 |
| Q3JRA0 | 3F0G | C5P | -6.25 | 3MBM | -6.38 | 0.13 |
| Q40577 | 5EAT | FHP | -7.57 | 5EAU | -7.42 | 0.15 |
| Q40577 | 5EAU | FFF | -7.57 | 5EAT | -8.37 | 0.80 |
| Q460N5 | 3SMI | QDR | -9.36 | 4F1L | -9.67 | 0.31 |
| Q460N5 | 4F1L | 0RY | -7.58 | 3SMI | -8.03 | 0.45 |
| Q46822 | 2VNP | DED | -6.77 | 1Q54 | -7.03 | 0.26 |
| Q46822 | 1Q54 | BHI | -7.57 | 2VNP | -7.75 | 0.18 |
| Q46893 | 1I52 | CTP | -9.76 | 1INI | -9.49 | 0.27 |
| Q46893 | 1INI | CDM | -9.63 | 1I52 | -9.22 | 0.41 |
| Q47066 | 4X69 | OP0 | -7.42 | 1IYO | -7.05 | 0.37 |
| Q47066 | 1IYO | CEF | -7.94 | 4X69 | -7.34 | 0.60 |
| Q47NQ8 | 4OMR | CAA | -8.01 | 4JVT | -6.41 | 1.60 |
| Q47NQ8 | 4JVT | ACO | -7.26 | 4OMR | -6.67 | 0.59 |
| Q4D3W2 | 3W85 | W85 | -8.94 | 3W7O | -8.87 | 0.07 |
| Q4D3W2 | 3W7O | W7O | -9.17 | 3W85 | -9.09 | 0.08 |
| Q4DA54 | 4YRR | 691 | -5.45 | 4YRM | -5.59 | 0.14 |
| Q4DA54 | 4YRM | 282 | -4.93 | 4YRR | -4.23 | 0.70 |
| Q4DA73 | 5Y4Q | 8OF | -9.52 | 4YUY | -8.07 | 1.45 |
| Q4DA73 | 4YUY | 1SQ | -7.22 | 5Y4Q | -6.94 | 0.28 |
| Q4QC75 | 4UXJ | TTP | -10.04 | 4UXH | -11.27 | 1.23 |
| Q4QC75 | 4UXH | T5A | -11.68 | 4UXJ | -10.62 | 1.06 |
| Q4W1X2 | 5VWT | FAD | -14.89 | 5HHF | -14.55 | 0.34 |
| Q4W1X2 | 5HHF | 62F | -15.80 | 5VWT | -12.46 | 3.34 |
| Q51504 | 3OCL | CB9 | -8.75 | 4FSF | -8.30 | 0.45 |
| Q51504 | 4FSF | 0W0 | -10.95 | 3OCL | -9.84 | 1.11 |
| Q52424 | 5US1 | COA | -8.55 | 5US1 | -7.67 | 0.88 |
| Q52424 | 5US1 | ACO | -7.98 | 5US1 | -8.19 | 0.21 |
| Q53752 | 3D4A | 3GP | -8.49 | 3DGY | -7.42 | 1.07 |
| Q53752 | 3DGY | 2GP | -7.51 | 3D4A | -7.46 | 0.05 |
| Q55891 | 2D1E | BLA | -13.59 | 3AJH | -9.67 | 3.92 |
| Q55891 | 3AJH | BL3 | -12.68 | 2D1E | -12.59 | 0.09 |
| Q57977 | 3BBH | SFG | -9.01 | 3BBD | -9.05 | 0.04 |
| Q57977 | 3BBD | SAH | -8.86 | 3BBH | -9.04 | 0.18 |
| Q58F21 | 5VBR | IBI | -7.99 | 4KCX | -8.19 | 0.20 |
| Q58F21 | 4KCX | 1QK | -7.86 | 5VBR | -7.71 | 0.15 |
| Q5A9A4 | 2WFG | ZZB | -11.39 | 5AGI | -11.41 | 0.02 |
| Q5A9A4 | 5AGI | ANZ | -10.98 | 2WFG | -11.11 | 0.13 |
| Q5AU62 | 1TI7 | NAP | -10.83 | 2VUT | -10.96 | 0.13 |
| Q5AU62 | 2VUT | NAD | -10.04 | 1TI7 | -9.56 | 0.48 |
| Q5G940 | 3CF9 | AGI | -8.85 | 3DOY | -7.26 | 1.59 |
| Q5G940 | 3DOY | 2BE | -9.11 | 3CF9 | -8.28 | 0.83 |
| Q5H3Z2 | 5CVQ | BB2 | -8.18 | 5CWY | -6.60 | 1.58 |
| Q5H3Z2 | 5CWY | 4WL | -6.30 | 5CVQ | -5.60 | 0.70 |
| Q5JIZ8 | 3WDL | ATP | -10.48 | 3WDM | -7.87 | 2.61 |
| Q5JIZ8 | 3WDM | ADN | -8.94 | 3WDL | -9.36 | 0.42 |
| Q5NFC4 | 3M5P | F6P | -7.32 | 3Q7I | -7.81 | 0.49 |
| Q5NFC4 | 3Q7I | 6PG | -8.09 | 3M5P | -6.66 | 1.43 |
| Q5SGX2 | 3HFZ | MTY | -8.43 | 1JJC | -8.39 | 0.04 |
| Q5SGX2 | 1JJC | FA5 | -11.93 | 3HFZ | -11.22 | 0.71 |
| Q5SHZ3 | 2ZDQ | ATP | -11.36 | 2ZDG | -8.87 | 2.49 |
| Q5SHZ3 | 2ZDG | ADP | -12.51 | 2ZDQ | -11.48 | 1.03 |
| Q5SLL8 | 4X3L | MTA | -9.22 | 4X3M | -8.86 | 0.36 |
| Q5SLL8 | 4X3M | ADN | -9.78 | 4X3L | -8.87 | 0.91 |
| Q5SQI0 | 4U9Z | COA | -10.64 | 4B5O | -10.27 | 0.37 |
| Q5SQI0 | 4B5O | ACO | -10.32 | 4U9Z | -9.57 | 0.75 |
| Q5TCY1 | 4BTM | F8E | -7.61 | 4BTJ | -7.36 | 0.25 |
| Q5TCY1 | 4BTJ | ATP | -9.51 | 4BTM | -8.82 | 0.69 |
| Q5TLG6 | 6D38 | GYC | -6.44 | 4HQC | -6.26 | 0.18 |
| Q5TLG6 | 4HQC | CR8 | -6.02 | 6D38 | -6.77 | 0.75 |
| Q5UQL3 | 3FBE | GDP | -9.23 | 3FC9 | -9.84 | 0.61 |
| Q5UQL3 | 3FC9 | CTP | -10.04 | 3FBE | -9.14 | 0.90 |
| Q5ZUA2 | 4BRL | GMV | -14.36 | 4BRF | -12.43 | 1.93 |
| Q5ZUA2 | 4BRF | AMP | -10.51 | 4BRL | -10.54 | 0.03 |
| Q5ZUA2 | 4BRH | TMV | -11.80 | 4BRG | -11.64 | 0.16 |
| Q5ZUA2 | 4BRG | GNP | -11.74 | 4BRH | -12.21 | 0.47 |
| Q62230 | 1OD7 | SUW | -7.78 | 1OD9 | -6.24 | 1.54 |
| Q62230 | 1OD9 | BND | -6.47 | 1OD7 | -6.45 | 0.02 |
| Q63T71 | 3IEW | CTP | -6.77 | 3IEW | -6.85 | 0.08 |
| Q63T71 | 3IEW | CDP | -6.47 | 3IEW | -6.64 | 0.17 |
| Q64610 | 5M0E | 5JK | -11.76 | 5DLW | -12.06 | 0.30 |
| Q64610 | 5DLW | 5D5 | -11.21 | 5M0E | -11.22 | 0.01 |
| Q6B856 | 5LP6 | 71P | -7.44 | 5JVD | -6.80 | 0.64 |
| Q6B856 | 5JVD | 6NL | -8.42 | 5LP6 | -8.34 | 0.08 |
| Q6DE08 | 4C2V | YJA | -9.03 | 4B8L | -8.29 | 0.74 |
| Q6DE08 | 4B8L | A0P | -11.32 | 4C2V | -10.59 | 0.73 |
| Q6FPH0 | 3EEM | 53V | -10.82 | 3EEK | -10.99 | 0.17 |
| Q6FPH0 | 3EEK | 53S | -11.46 | 3EEM | -11.28 | 0.18 |
| Q6N089 | 3QCU | NKN | -6.16 | 3QCV | -5.86 | 0.30 |
| Q6N089 | 3QCV | 18L | -6.03 | 3QCU | -5.99 | 0.04 |
| Q6N5P6 | 2I4O | ATP | -12.08 | 2I4N | -11.31 | 0.77 |
| Q6N5P6 | 2I4N | 5CA | -11.00 | 2I4O | -10.46 | 0.54 |
| Q6P179 | 5K1V | 6PX | -9.64 | 5J6S | -10.00 | 0.36 |
| Q6P179 | 5J6S | 6GA | -10.58 | 5K1V | -10.28 | 0.30 |
| Q6PL18 | 4TYL | 39O | -5.49 | 4TU4 | -5.92 | 0.43 |
| Q6PL18 | 4TU4 | 37N | -8.15 | 4TYL | -7.69 | 0.46 |
| Q6VT83 | 6AWR | 2AN | -6.20 | 4MA6 | -6.69 | 0.49 |
| Q6VT83 | 4MA6 | 28E | -8.75 | 6AWR | -6.78 | 1.97 |
| Q6YMS4 | 5JJS | 6L2 | -9.18 | 5F3T | -8.38 | 0.80 |
| Q6YMS4 | 5F3T | 5UH | -7.49 | 5JJS | -7.71 | 0.22 |
| Q74FS9 | 4GVL | AMP | -9.42 | 4GX1 | -9.84 | 0.42 |
| Q74FS9 | 4GX1 | ADP | -9.00 | 4GVL | -9.07 | 0.07 |
| Q79SH7 | 3HTH | PRL | -10.82 | 3HTJ | -10.54 | 0.28 |
| Q79SH7 | 3HTJ | ET | -13.84 | 3HTH | -7.09 | 6.75 |
| Q7BG50 | 2X0E | TYD | -8.38 | 2X0F | -8.83 | 0.45 |
| Q7BG50 | 2X0F | TRH | -11.24 | 2X0E | -10.31 | 0.93 |
| Q7K4Y6 | 4XPH | 42J | -7.80 | 4XP9 | -6.51 | 1.29 |
| Q7K4Y6 | 4XP9 | 1WE | -6.27 | 4XPH | -7.13 | 0.86 |
| Q7M537 | 4B8S | AMP | -8.62 | 1GC5 | -8.02 | 0.60 |
| Q7M537 | 1GC5 | ADP | -9.22 | 4B8S | -9.33 | 0.11 |
| Q7Z1V1 | 4CK8 | LFD | -9.79 | 4C27 | -9.66 | 0.13 |
| Q7Z1V1 | 4C27 | 26N | -11.31 | 4CK8 | -11.48 | 0.17 |
| Q81R22 | 3FL8 | RAR | -11.84 | 3JWF | -9.14 | 2.70 |
| Q81R22 | 3JWF | 5WA | -8.43 | 3FL8 | -7.60 | 0.83 |
| Q831W7 | 2J3L | P5A | -13.03 | 2J3M | -12.52 | 0.51 |
| Q831W7 | 2J3M | ATP | -11.70 | 2J3L | -11.12 | 0.58 |
| Q83XK4 | 2XLS | NAP | -9.97 | 2XLU | -9.58 | 0.39 |
| Q83XK4 | 2XLU | NA7 | -9.17 | 2XLS | -9.40 | 0.23 |
| Q86W56 | 4B1I | A8P | -10.45 | 4B1J | -9.37 | 1.08 |
| Q86W56 | 4B1J | A1R | -11.77 | 4B1I | -10.82 | 0.95 |
| Q86X55 | 2Y1W | 849 | -10.16 | 5U4X | -9.58 | 0.58 |
| Q86X55 | 5U4X | 7VM | -9.80 | 2Y1W | -9.34 | 0.46 |
| Q873X9 | 2IUZ | D1H | -8.12 | 2A3E | -8.38 | 0.26 |
| Q873X9 | 2A3E | AMI | -6.58 | 2IUZ | -6.17 | 0.41 |
| Q89VT8 | 4H2W | AMP | -10.19 | 4H2W | -9.48 | 0.71 |
| Q89VT8 | 4H2W | 5GP | -9.52 | 4H2W | -10.18 | 0.66 |
| Q89ZI2 | 2WCA | NP6 | -8.86 | 2J47 | -8.85 | 0.01 |
| Q89ZI2 | 2J47 | GDV | -9.07 | 2WCA | -8.02 | 1.05 |
| Q8GIQ0 | 2IXL | TRH | -7.86 | 1NYW | -8.40 | 0.54 |
| Q8GIQ0 | 1NYW | DAU | -8.68 | 2IXL | -7.90 | 0.78 |
| Q8II73 | 3B7P | SPM | -5.84 | 4BP3 | -5.65 | 0.19 |
| Q8II73 | 4BP3 | 4MN | -6.50 | 3B7P | -5.80 | 0.7 |
| Q8IXJ6 | 5G4C | CNA | -14.62 | 4RMJ | -13.67 | 0.95 |
| Q8IXJ6 | 4RMJ | AR6 | -13.81 | 5G4C | -12.81 | 1.00 |
| Q8KI25 | 3EPT | FDA | -14.19 | 2R0P | -11.84 | 2.35 |
| Q8KI25 | 2R0P | FAD | -13.79 | 3EPT | -13.47 | 0.32 |
| Q8N8S7 | 5NEG | 8VK | -8.98 | 4MY6 | -8.30 | 0.68 |
| Q8N8S7 | 4MY6 | 3VH | -8.91 | 5NEG | -9.15 | 0.24 |
| Q8NKB0 | 3WZM | ZER | -11.27 | 5IE4 | -11.45 | 0.18 |
| Q8NKB0 | 5IE4 | 36J | -11.87 | 3WZM | -10.76 | 1.11 |
| Q8PC69 | 2JLB | UDM | -11.18 | 2XGS | -9.83 | 1.35 |
| Q8PC69 | 2XGS | 44P | -9.04 | 2JLB | -9.44 | 0.40 |
| Q8TEK3 | 5MW4 | 5JU | -11.43 | 5DTQ | -11.03 | 0.40 |
| Q8TEK3 | 5DTQ | 5F6 | -11.29 | 5MW4 | -7.88 | 3.41 |
| Q8WWQ0 | 5ENC | 5QD | -5.55 | 5ENE | -5.83 | 0.28 |
| Q8WWQ0 | 5ENE | 5Q8 | -6.64 | 5ENC | -6.66 | 0.02 |
| Q8YUQ7 | 3L6J | Z90 | -11.38 | 4JQH | -10.61 | 0.77 |
| Q8YUQ7 | 4JQH | 1MF | -12.98 | 3L6J | -12.21 | 0.77 |
| Q92769 | 4LXZ | SHH | -7.34 | 6G3O | -6.95 | 0.39 |
| Q92769 | 6G3O | EL8 | -7.44 | 4LXZ | -7.39 | 0.05 |
| Q92830 | 5TRL | SCA | -8.13 | 1Z4R | -7.56 | 0.57 |
| Q92830 | 1Z4R | ACO | -7.64 | 5TRL | -8.48 | 0.84 |
| Q93IG4 | 2JAS | DTP | -10.45 | 2JAQ | -10.80 | 0.35 |
| Q93IG4 | 2JAQ | DCP | -11.40 | 2JAS | -9.91 | 1.49 |
| Q93PQ0 | 3BFF | SFR | -7.41 | 3BFC | -7.35 | 0.06 |
| Q93PQ0 | 3BFC | IM2 | -7.09 | 3BFF | -6.65 | 0.44 |
| Q965D7 | 3AZA | KM0 | -6.49 | 3AZ9 | -6.70 | 0.21 |
| Q965D7 | 3AZ9 | K91 | -6.71 | 3AZA | -7.07 | 0.36 |
| Q96C86 | 3BL7 | DD1 | -9.88 | 5OSY | -9.42 | 0.46 |
| Q96C86 | 5OSY | AJQ | -10.98 | 3BL7 | -11.08 | 0.10 |
| Q96PN6 | 5IV3 | LRI | -8.74 | 4OYA | -6.90 | 1.84 |
| Q96PN6 | 4OYA | 1VE | -11.03 | 5IV3 | -9.96 | 1.07 |
| Q96RI1 | 5Q0J | 9KV | -13.70 | 5Q0X | -12.30 | 1.40 |
| Q96RI1 | 5Q17 | 9MV | -11.44 | 5Q0J | -8.27 | 3.17 |
| Q96RI1 | 5Q14 | 9MM | -13.07 | 5Q14 | -12.72 | 0.35 |
| Q96RI1 | 5Q0X | 643 | -12.72 | 5Q17 | -10.01 | 2.71 |
| Q97ZE2 | 2J4J | ACP | -10.84 | 2J4J | -10.77 | 0.07 |
| Q97ZE2 | 2J4J | 4TC | -14.03 | 2J4J | -12.54 | 1.49 |
| Q980Q4 | 1VST | PRP | -8.42 | 3G6W | -8.44 | 0.02 |
| Q980Q4 | 3G6W | HSX | -7.28 | 1VST | -7.32 | 0.04 |
| Q98SW5 | 5KZV | HCD | -10.51 | 5KZY | -9.46 | 1.05 |
| Q98SW5 | 5KZY | CY8 | -12.30 | 5KZV | -12.13 | 0.17 |
| Q99418 | 4JMO | JAF | -7.07 | 4L5M | -7.20 | 0.13 |
| Q99418 | 4L5M | HRC | -7.06 | 4JMO | -6.68 | 0.38 |
| Q9AIU0 | 2UXI | G50 | -7.82 | 2UXP | -7.90 | 0.08 |
| Q9AIU0 | 2UXP | CLM | -6.85 | 2UXI | -6.75 | 0.10 |
| Q9BH77 | 2OP1 | 8PC | -10.24 | 2OP0 | -9.43 | 0.81 |
| Q9BH77 | 1NHW | TCC | -7.68 | 2OP1 | -8.48 | 0.80 |
| Q9BH77 | 2OP0 | 7PC | -9.71 | 2OP1 | -10.06 | 0.35 |
| Q9BH77 | 2OP1 | 8PC | -10.24 | 1NHW | -8.95 | 1.29 |
| Q9BQ65 | 5V1M | U5P | -7.60 | 6D31 | -7.64 | 0.04 |
| Q9BQ65 | 6D31 | A | -7.79 | 5V1M | -8.06 | 0.27 |
| Q9BY41 | 4RN0 | L6G | -6.32 | 3SFH | -6.46 | 0.14 |
| Q9BY41 | 3SFH | 1DI | -10.37 | 4RN0 | -7.46 | 2.91 |
| Q9BYW2 | 5JLB | SAH | -9.91 | 5LSX | -10.35 | 0.44 |
| Q9BYW2 | 5LSX | 76O | -12.29 | 5JLB | -11.95 | 0.34 |
| Q9BZP6 | 3RM8 | RM8 | -10.62 | 3RM4 | -9.22 | 1.40 |
| Q9BZP6 | 3RM4 | 3RM | -8.85 | 3RM8 | -7.91 | 0.94 |
| Q9F663 | 3RXX | NPB | -6.20 | 6B1J | -7.11 | 0.91 |
| Q9F663 | 6B1J | C8V | -8.88 | 3RXX | -8.05 | 0.83 |
| Q9GPQ4 | 2FF2 | IMH | -10.20 | 1HP0 | -10.62 | 0.42 |
| Q9GPQ4 | 1HP0 | AD3 | -10.17 | 2FF2 | -9.69 | 0.48 |
| Q9GZ28 | 1XMZ | CRK | -5.89 | 1XQM | -5.88 | 0.01 |
| Q9GZ28 | 1XQM | CH6 | -6.58 | 1XMZ | -6.26 | 0.32 |
| Q9H0U4 | 5SZH | GNP | -14.10 | 4I1O | -12.29 | 1.81 |
| Q9H0U4 | 4I1O | GDP | -11.65 | 5SZH | -11.76 | 0.11 |
| Q9H2K2 | 5FPG | Q28 | -15.92 | 4W5I | -11.95 | 3.97 |
| Q9H2K2 | 4W5I | 3GX | -11.49 | 5FPG | -10.34 | 1.15 |
| Q9H4A3 | 5WE8 | A7Y | -11.77 | 5TF9 | -10.41 | 1.36 |
| Q9H4A3 | 5TF9 | 7AV | -10.48 | 5WE8 | -10.04 | 0.44 |
| Q9H8M2 | 4Z6H | 4L2 | -8.85 | 4XY8 | -8.27 | 0.58 |
| Q9H8M2 | 4XY8 | 43U | -7.92 | 4Z6H | -7.52 | 0.40 |
| Q9H8M2 | 5TWX | 7P7 | -8.58 | 5E9V | -7.87 | 0.71 |
| Q9H8M2 | 5E9V | 5L0 | -9.17 | 5TWX | -8.97 | 0.20 |
| Q9H9B1 | 3MO2 | E67 | -8.11 | 3MO0 | -7.39 | 0.72 |
| Q9H9B1 | 3MO0 | E11 | -6.69 | 3MO2 | -7.48 | 0.79 |
| Q9HU22 | 4B42 | 942 | -6.73 | 3ZLL | -7.08 | 0.35 |
| Q9HU22 | 3ZLL | 4WF | -7.90 | 4B42 | -7.96 | 0.06 |
| Q9K2N0 | 5LCH | 6TU | -9.45 | 5LCF | -8.29 | 1.16 |
| Q9K2N0 | 5LCF | 6TJ | -9.68 | 5LCH | -8.69 | 0.99 |
| Q9KTW3 | 4QNE | NAJ | -10.69 | 4X3Z | -11.11 | 0.42 |
| Q9KTW3 | 4X3Z | NAD | -11.45 | 4QNE | -10.78 | 0.67 |
| Q9KU37 | 2OXN | OAN | -7.11 | 3GS6 | -6.82 | 0.29 |
| Q9KU37 | 3GS6 | NP6 | -7.11 | 2OXN | -7.03 | 0.08 |
| Q9M9P3 | 2ICX | UTP | -9.77 | 2ICY | -9.33 | 0.44 |
| Q9M9P3 | 2ICY | UPG | -10.50 | 2ICX | -10.88 | 0.38 |
| Q9NAV7 | 5LK9 | LZ1 | -5.60 | 5LLZ | -5.38 | 0.22 |
| Q9NAV7 | 5LLZ | 0CT | -5.27 | 5LK9 | -5.29 | 0.02 |
| Q9NAV8 | 4HSX | BML | -5.68 | 3LB3 | -5.12 | 0.56 |
| Q9NAV8 | 3LB3 | 4CH | -4.98 | 4HSX | -5.48 | 0.50 |
| Q9NPB1 | 4MWO | 2E02 | -12.12 | 4MWO | -11.46 | 0.66 |
| Q9NPB1 | 4YIK | 2O2 | -11.40 | 4MWO | -10.53 | 0.87 |
| Q9NPB1 | 4MWO | 2E02 | -12.12 | 4NFL | -11.12 | 1.00 |
| Q9NPB1 | 4NFL | 2JW | -11.10 | 4YIK | -10.46 | 0.64 |
| Q9NR97 | 4R07 | UCG | -10.15 | 4R09 | -9.45 | 0.70 |
| Q9NR97 | 4R09 | 06S | -9.37 | 4R07 | -9.02 | 0.35 |
| Q9NRW1 | 2FFQ | GSP | -13.44 | 2FE4 | -12.00 | 1.44 |
| Q9NRW1 | 2FE4 | GDP | -11.84 | 2FFQ | -11.99 | 0.15 |
| Q9NWT6 | 4AI8 | DZA | -5.16 | 5OP8 | -4.32 | 0.84 |
| Q9NWT6 | 5OP8 | A1H | -8.12 | 4AI8 | -8.38 | 0.26 |
| Q9NZJ5 | 4X7K | 3Z3 | -15.48 | 4X7J | -14.45 | 1.03 |
| Q9NZJ5 | 4X7J | 3Z1 | -13.07 | 4X7K | -12.45 | 0.62 |
| Q9QLL6 | 5EFA | MGT | -7.48 | 5EFC | -7.22 | 0.26 |
| Q9QLL6 | 5EFC | GTP | -8.65 | 5EFA | -8.60 | 0.05 |
| Q9R194 | 4I6G | FAD | -12.71 | 4MLP | -11.24 | 1.47 |
| Q9R194 | 4MLP | 2CX | -10.24 | 4I6G | -9.70 | 0.54 |
| Q9R1E6 | 5OLB | 6ZO | -10.63 | 5LIA | -10.85 | 0.22 |
| Q9R1E6 | 5LIA | 6XN | -12.39 | 5OLB | -10.75 | 1.64 |
| Q9RA63 | 4LJ7 | MNT | -9.62 | 4FD2 | -10.32 | 0.70 |
| Q9RA63 | 4FD2 | ADP | -10.41 | 4LJ7 | -9.85 | 0.56 |
| Q9RC92 | 2AHG | UCD | -8.91 | 2FV1 | -8.55 | 0.36 |
| Q9RC92 | 2FV1 | GAD | -6.63 | 2AHG | -7.48 | 0.85 |
| Q9UGL1 | 5FZL | UOI | -8.02 | 5A3T | -7.60 | 0.42 |
| Q9UGL1 | 5A3T | MMK | -8.29 | 5FZL | -8.20 | 0.09 |
| Q9UHI6 | 3B7G | ANP | -8.64 | 2OXC | -8.60 | 0.04 |
| Q9UHI6 | 2OXC | ADP | -8.27 | 3B7G | -7.42 | 0.85 |
| Q9UJ71 | 5G6U | YJM | -6.04 | 4N32 | -5.83 | 0.21 |
| Q9UJ71 | 4N32 | 2F8 | -4.99 | 5G6U | -4.93 | 0.06 |
| Q9UKV8 | 3QX9 | ATP | -6.98 | 3LUD | -6.82 | 0.16 |
| Q9UKV8 | 3LUD | AMP | -7.12 | 3QX9 | -7.17 | 0.05 |
| Q9UM73 | 4FNZ | NZF | -11.16 | 4FOB | -8.68 | 2.48 |
| Q9UM73 | 4FOB | 0US | -9.62 | 4FNZ | -8.39 | 1.23 |
| Q9UVX3 | 4CAX | 646 | -9.82 | 4CAV | -9.59 | 0.23 |
| Q9UVX3 | 4CAV | 2XQ | -10.84 | 4CAX | -9.06 | 1.78 |
| Q9UVX3 | 4CAW | P3U | -11.10 | 5T5U | -10.22 | 0.88 |
| Q9UVX3 | 5T5U | 75T | -11.44 | 4CAW | -10.88 | 0.56 |
| Q9UYR9 | 1YR7 | GSP | -9.64 | 1YRB | -9.99 | 0.35 |
| Q9UYR9 | 1YRB | GDP | -10.01 | 1YR7 | -9.28 | 0.73 |
| Q9V2G1 | 5HJK | SAH | -9.86 | 5HJM | -10.34 | 0.48 |
| Q9V2G1 | 5HJM | MTA | -9.46 | 5HJK | -9.00 | 0.46 |
| Q9WUL6 | 5T8Q | 76Y | -6.49 | 4G3E | -7.04 | 0.55 |
| Q9WUL6 | 4G3E | 0WC | -11.48 | 5T8Q | -8.75 | 2.73 |
| Q9X1X0 | 4O5G | 2PV | -7.84 | 4O4K | -6.28 | 1.56 |
| Q9X1X0 | 4O4K | 2PK | -8.39 | 4O5G | -7.87 | 0.52 |
| Q9Y3R4 | 2F11 | IEM | -6.65 | 1VCU | -7.29 | 0.64 |
| Q9Y3R4 | 1VCU | DAN | -7.88 | 2F11 | -6.79 | 1.09 |
| Q9Y3Z3 | 6DWK | HFD | -10.10 | 4TNY | -9.86 | 0.24 |
| Q9Y3Z3 | 4TNY | DGT | -10.32 | 6DWK | -10.32 | 0.00 |
| Q9Y6E0 | 4QO9 | 627 | -9.45 | 4QMS | -8.57 | 0.88 |
| Q9Y6E0 | 4QMS | 1N1 | -9.14 | 4QO9 | -7.75 | 1.39 |
| Q9ZAG3 | 5YQT | 3ZS | -3.95 | 5JPU | -3.98 | 0.03 |
| Q9ZAG3 | 5JPU | 3ZQ | -4.89 | 5YQT | -5.45 | 0.56 |
| U6AVY0 | 4OOM | 2U3 | -9.50 | 4OOL | -9.83 | 0.33 |
| U6AVY0 | 4OOL | 2U2 | -8.91 | 4OOM | -9.03 | 0.12 |
